# Supplementary material for: Assessing community engagement in Nigeria polio eradication initiative: application of the Consolidated Framework for Implementation Research
Source: BMJ Open. 2021 Aug 9;11(8):e048694. doi: 10.1136/bmjopen-2021-048694 (PMC8354285; doi:10.1136/bmjopen-2021-048694)
Supplement: Author's manuscript [file bmjopen-2021-048694.draft_revisions.pdf]

BMJ Open is committed to open peer review. As part of this commitment we make the peer review history of every article we publish publicly available.

When an article is published we post the peer reviewers' comments and the authors' responses online. We also post the versions of the paper that were used during peer review. These are the versions that the peer review comments apply to.

The versions of the paper that follow are the versions that were submitted during the peer review process. They are not the versions of record or the final published versions. They should not be cited or distributed as the published version of this manuscript.

BMJ Open is an open access journal and the full, final, typeset and author-corrected version of record of the manuscript is available on our site with no access controls, subscription charges or pay-per-view fees (http://bmjopen.bmj.com).

If you have any questions on BMJ Open's open peer review process please email info.bmjopen@bmj.com

BMJ Open

Assessing community engagement in Nigeria polio eradication initiative: application of the Consolidated Framework for Implementation Research

Journal:	*BMJ Open*
Manuscript ID	bmjopen-2021-048694
Article Type:	Original research
Date Submitted by the Author:	05-Jan-2021

Complete List of Authors:	Akinyemi, Oluwaseun; University of Ibadan College of Medicine, Health Policy and Management<br>Adebayo, Adedamola; University of Ibadan College of Medicine, Department of Community Medicine<br>Bassey, Christopher ; University of Ibadan College of Medicine, Department of Community Medicine<br>Nwaiwu, Chioma ; University of Ibadan College of Medicine, Department of Community Medicine<br>Kalbarczyk, Anna; Johns Hopkins University Bloomberg School of Public Health, International Health<br>Fatiregun, Akinola; World Health Organization Country Office for Nigeria, Ondo State Field Office<br>Alonge, Olakunle; Johns Hopkins University Bloomberg School of Public Health,<br>Owoaje, Eme; University of Ibadan College of Medicine, Community Medicine

Keywords:	Public health < INFECTIOUS DISEASES, Health policy < HEALTH SERVICES ADMINISTRATION & MANAGEMENT, Paediatric infectious disease & immunisation < PAEDIATRICS

SCHOLARONE™
Manuscripts

I, the Submitting Author has the right to grant and does grant on behalf of all authors of the Work (as defined in the below author licence), an exclusive licence and/or a non-exclusive licence for contributions from authors who are: i) UK Crown employees; ii) where BMJ has agreed a CC-BY licence shall apply, and/or iii) in accordance with the terms applicable for US Federal Government officers or employees acting as part of their official duties; on a worldwide, perpetual, irrevocable, royalty-free basis to BMJ Publishing Group Ltd ("BMJ") its licensees and where the relevant Journal is co-owned by BMJ to the co-owners of the Journal, to publish the Work in this journal and any other BMJ products and to exploit all rights, as set out in our licence.

The Submitting Author accepts and understands that any supply made under these terms is made by BMJ to the Submitting Author unless you are acting as an employee on behalf of your employer or a postgraduate student of an affiliated institution which is paying any applicable article publishing charge ("APC") for Open Access articles. Where the Submitting Author wishes to make the Work available on an Open Access basis (and intends to pay the relevant APC), the terms of reuse of such Open Access shall be governed by a Creative Commons licence – details of these licences and which Creative Commons licence will apply to this Work are set out in our licence referred to above.

Other than as permitted in any relevant BMJ Author's Self Archiving Policies, I confirm this Work has not been accepted for publication elsewhere, is not being considered for publication elsewhere and does not duplicate material already published. I confirm all authors consent to publication of this Work and authorise the granting of this licence.

For peer review only

Assessing community engagement in Nigeria polio eradication initiative: application of the Consolidated Framework for Implementation Research

Oluwaseun Akinyemi,[a*] (ooakinyemi@com.ui.edu.ng, seunakinyemi@hotmail.com)

Adedamola Adebayo,[b] (adewalededamola@gmail.com)

Christopher Bassey,[b] (christopher.bassey@gmail.com)

Chioma Nwaiwu,[b] (chiomanwaiwu71@yahoo.com)

Anna Kalbarczyk,[c] (akalbarc@jhu.edu)

Akinola Fatiregun,[d] (akinfati@yahoo.com)

Olakunle O. Alonge, [c] (oalonge1@jhu.edu)

Eme T. Owoaje, [b] (emetheodora@gmail.com)

[a] *Department of Health Policy and Management, College of Medicine, University of Ibadan, Ibadan, Nigeria*

[b] *Department of Community Medicine, College of Medicine, University of Ibadan, Ibadan, Nigeria*

[c] *Johns Hopkins Bloomberg School of Public Health, Baltimore, USA*

[d] *World Health Organization, Ondo State Field Office, Nigeria*

Original research article

***Corresponding author:** Dr Oluwaseun Akinyemi

Department of Health Policy and Management

College of Medicine, University of Ibadan, Ibadan, Nigeria

+234 803 502 0136

Keywords: Polio eradication initiative, Implementation science, Health policy analysis, Community participation

Word counts:

Abstract: 272

Manuscript: 4640

For peer review only

Assessing community engagement in Nigeria polio eradication initiative: application of the Consolidated Framework for Implementation Research

Abstract

Objective To assess factors that enhanced or impeded the implementation of community engagement strategies using the Nigerian polio program as a point of reference.

Design This study was a part of a larger descriptive cross-sectional survey. Consolidated Framework for Implementation Research (CFIR) framework was utilized to design the instrument which was administered through face-to-face and phone interviews and a web-based data collection platform, Qualtrics.

Setting The study took place in at least one State from each of the six geopolitical zones in Nigeria as well as the Federal Capital Territory.

Participants The respondents included program managers, policymakers, researchers and frontline field implementers affiliated with the Global Polio Eradication Initiative (GPEI) core partner organizations, the three tiers of the government health parastatals (local, state and federal levels) and academic/research institutions.

Results Data for this study were obtained from 364 respondents who reported participation in community engagement activities in Nigeria's PEI. Majority (68.4%) had less than 10 years' experience in PEI, 57.4% were involved at the local government level and 46.9% were team supervisors. Almost half (45.0%) of the participants identified the process of conducting the PEI program and social environment (56.0%) as the most important internal and external contributor to implementing community engagement activities in the community, respectively. The economic environment (35.7%) was the most frequently reported challenge among the external challenges to implementing community engagement activities.

Conclusion Community engagement strategies were largely affected by factors relating to the process of conducting the polio program, the economic environment and the social context. Therefore, community engagement implementers should focus on these key areas and channel resources to reduce obstacles to achieve community engagement goals.

For peer review only - http://bmjopen.bmj.com/site/about/guidelines.xhtml

Strengths and limitations of this study

- This study presents findings about one of the most important strategy of polio eradication program and presented findings which are useful for public health program implementers in achieving community engagement objectives
- The participants in this study are a representative of all categories of stakeholders from the national to the local levels that participated in the polio program thereby bringing in their nuanced insights.
- Social desirability bias was a possibility because participants were involved in the program and would probably not be inclined to present the activities in a negative light.
- This study utilized a cross-sectional design therefore it was impossible to determine causal relationships between variables. Furthermore, there was a tendency to recall bias as the PEI spanned a few decades.

Keywords: Community engagement, Polio eradication initiative, Consolidated framework for implementation research, Implementation science

Funding

This study is funded by the Bill & Melinda Gates Foundation (OPP1178578, Teaching Global Health Leaders about the Lessons Learned from Polio Eradication). The funder did not play any role in writing the protocol, interpreting the data, or in writing this manuscript. The funder provided and coordinated external peer-review for the study proposal. Publication costs were funded by the Bill and Melinda Gates Foundation.

Competing interests

The authors declare that they have no competing interests.

For peer review only - http://bmjopen.bmj.com/site/about/guidelines.xhtml

Introduction

Community engagement is a fundamental principle in the planning, organizing and delivery of primary health care services to the community.[1, 2] This principle, set during the Alma-Ata declaration of 1978, remains relevant in the Sustainable Development Goals (SDG) era when engaging the community is the priority for the achievement of universal health coverage by 2030.[3] The benefits of engaging the community in the planning and implementation phases of health interventions have been documented across various maternal and child health programs, including the polio eradication program.[4-6] In the primary health care system, this strategy supports relationship building with community members,[7] reduces health inequalities,[8] provides a channel for the dissemination of information on health products and services and aids the health system to adapt the health service to the local context and address misinformation about health interventions.[9, 10]

The implementation of community engagement strategies in the polio program has enhanced coverage, acceptability and ownership of health programs in high-risk areas such as hard-to-reach and conflict-affected communities, which would have otherwise been difficult to achieve.[5] Community engagement has been beneficial, and has encouraged the adoption of health services by individuals who have negative beliefs and attitudes by engaging them to discuss the basis of their beliefs and encouraging them to proffer solutions to issues they identified.[11] One successful example of community engagement in high-risk areas includes the 2014 engagement of 11,000 female community-based mobilizers in Nigeria to advocate for vaccine uptake in northern Nigeria.[12] This resulted in over 322,000 newborns referred for routine immunization and over 32,000 malnourished children referred for appropriate care.[12]

Alternatively, when community engagement was not used and these high-risk communities were neglected during planning and implementation of public health interventions, misconceptions about the program emerged leading to noncompliance to the public health directives or outright rejection of health interventions.[13] The polio program in Nigeria has had firsthand experience with vaccine hesitancy and rejection of the polio vaccines. This was a result of neglecting political and religious leaders' opinions and not addressing their misconceptions before program implementation, especially in Northern Nigeria.[13, 14] This challenge was eventually resolved through dialogue with religious and political leaders and by engaging the community in

For peer review only - http://bmjopen.bmj.com/site/about/guidelines.xhtml

validating the vaccine.[6] The Global Polio Eradication Initiative (GPEI) then adopted this strategy in addressing other challenges that arose in these communities and in other countries yet to be certified polio-free.[15-17] The community engagement strategy has taken various formats over the years ranging from engaging religious leaders and traditional leaders to engaging opinion leaders and other members of the community. Other community engagement strategies include creating media awareness as well as providing incentives to caregivers and other participating community members. These innovations have yielded substantial benefits to the immunization system and the primary health care system.[4, 18]

Rationale and Objectives

The wild polio virus was last detected in Nigeria in 2016, and community engagement has been identified as a principal strategy for achieving this success.[12] However, as most community engagement activities in polio have been reactionary, there is a gap in our understanding of how to systematically translate best practices in community engagement activities to other programs and contexts. Whereas other Primary Health Care programs have leveraged on the gains of the polio eradication initiative in Nigeria and are adopting the strategies employed by the polio program, including community engagement. The adoption of these strategies has yielded mixed results due variable implementation – and lack of a systematic model to adapt these strategies to other contexts. Therefore, there is a need to extensively discuss potential factors that could hinder or facilitate the implementation of community engagement strategies using the polio program in Nigeria as a case study. The Consolidated Framework for Implementation Research (CFIR)[19] provides relevant domains and constructs for analyzing public health strategies, can be applied to systematically identify relevant aspects of implementation of community engagement strategy that need to be addressed for the strategy to be successful in any context. This study used the CFIR framework to identify factors that facilitated or hindered the implementation of community engagement activities within the Nigeria's Polio Eradication Initiative (PEI). The study was conducted as part of a multi-country implementation science project, Synthesis and Translation of Research and Innovations from Polio Eradication (STRIPE), to capture lesson learned from the Global Polio Eradication Initiative (GPEI).[20] It is hoped that the knowledge of these factors will enhance the adaptation of community engagement activities in other public health programs, and the effective delivery of these programs in Nigeria and similar settings.

For peer review only - http://bmjopen.bmj.com/site/about/guidelines.xhtml

Methods

Study setting

Nigeria is the most populous country in Sub-Saharan Africa with an estimated population of 200 million persons of which over two-thirds are children and young persons.[21] Nigeria is divided into six geopolitical zones - South-South, South-East, South-West, North-East, North-Central and North-West. Overall, Northwest, Kano-Sokoto hub, have the highest number of cases followed by the Northeast, Borno -Yobe hub.[22]

Nigeria has been experiencing security challenges initially from the militants in the south and insurgents and armed bandits in the northern part of the country. This has resulted in multiple attempts to attack health workers engaged in vaccination efforts.[23, 24] Consequently, the country missed the polio-free certification in 2016 when a case of polio was reported in the North East region (Borno), one of the areas affected by conflict and insecurity.[18] However, it has been three years since the last case of the wild poliovirus was identified in Nigeria[25] and the country was eventually declared polio-free by the WHO in June 2020.[26]

Study design and participants

This research was a part of a larger descriptive cross-sectional survey that utilized the CFIR as an evaluation framework to identify critical challenges and contributors in the implementation of community engagement activities within the PEI in Nigeria.

The Consolidated Framework for Implementation Research (CFIR)[19] is an evaluation tool for assessing implementation activities, challenges and contributors. The CFIR was selected because it is a multi-theoretical framework that has been used to synthesize research evidence from various disciplines into a consolidated framework with multiple constructs of what works, where and why across various contexts.[19, 27] The framework has 39 constructs organized into five major domains found to influence the successful implementation of innovative programs. The domains assess include: i) Characteristics of the innovation; ii) Outer setting including the economic, political and social context; iii) Inner setting including organizational culture and climate; iv) Characteristics of individuals involved in implementation and v) Process (i.e. methods by which the intervention was conducted).[27]

For peer review only - http://bmjopen.bmj.com/site/about/guidelines.xhtml

The respondents were eligible to participate in the study based on their engagement in PEI activities for at least one year since the inception in 1988. The respondents included program managers, policymakers, researchers and frontline field implementers affiliated with the GPEI core partner organizations, the three tiers of the government health parastatals and academic/research institutions. The respondents were identified across the three tiers of government in Nigeria – federal, state (sub-national) and local government (district). At the federal level, key stakeholders in the PEI were recruited from the National Primary Healthcare Development Agency (NPHCDA), Federal Ministry of Health and GPEI core partners group including WHO, UNICEF and Bill and Melinda Gates Foundation. At the state (sub-national) level, Polio Eradication Initiative (PEI) managers, supervisors, team leads, facilitators were recruited. At the local government (district) level, frontline implementers in the polio program were recruited.

Sampling strategy

To have representation from the six geopolitical zones in Nigeria, at least one state but not more than three states were purposively selected from each zone. Participants were selected from Nasarawa state and Federal Capital Territory in the North-Central zone; Borno state in the North-East zone; Kano and Sokoto states in the North-West zone; Anambra state in the South-East zone; Bayelsa state in the South-South zone; and Lagos, Ondo and Oyo states in the South-West zone. At the federal level, key stakeholders in the polio eradication initiative were contacted from the National Primary Healthcare Development agency (NPHCDA), Ministry of Health and GPEI core partners group. At the sub national and district level, Polio Eradication Initiative (PEI) mangers, supervisors, team leads, facilitators were contacted. While at the local level, front line field implementers in the polio program. In total, 998 respondents participated in the survey but only 966 responses were recorded valid and complete and used in the analysis of the study. From the 966 respondents, a preliminary analysis was conducted, findings show that 364 participants had been involved in community engagement activities throughout their involvement with the polio program. This subset of response was used in developing this paper.

Recruitment process

The participants were recruited through emails, telephone calls and in-persons contact. This means of recruitment was used to ensure the estimated sample of 1000 participants was

For peer review only - http://bmjopen.bmj.com/site/about/guidelines.xhtml

achieved. The research team approached potential participants through the States Ministry of Health, the contact persons within the states provided list of health workers who had participated in polio program. This list includes State Immunization Officers, Surveillance Officers, Technical facilitators involved in the polio eradication programme.

Survey instrument

Development of the survey instrument was informed by activities of the PEI program, tools used by the GPEI partners to generate lessons learned from the polio program and attempts to fill gaps left from previous efforts as well as external efforts. Key GPEI program activities domains (and related implementation challenges) were identified in a literature review of GPEI literature.

The survey instrument was a structured closed questionnaire with sections on demographics and a section on participants' experiences with implementing community engagement activities. The participants' experiences implementing community engagement activities was organized into two categories:

1: Factors that contributed to the success in implementation of community engagement activities in the PEI, Nigeria (Facilitators).

2: Factors that impeded the implementation of community engagement activities in the PEI, Nigeria (Barriers).

For the two categories, the CFIR framework was used to identify the domains and associated constructs which contributed to success or challenges in implementing community engagement activities.

[Table 1: CFIR domains and associated constructs]

For peer review only - http://bmjopen.bmj.com/site/about/guidelines.xhtml

Survey pilot

This survey was pretested among 10 resident doctors at the College of Medicine that had been involved in polio eradication programme in Oyo state. These respondents provided face content and validity for the questionnaires. A few ambiguous questions were then rephrased following the pretest.

Data collection procedures

The survey instrument was administered online using Qualtrics, a web-based data collection and analysis platform. The self-administered survey was in English language. The Qualtrics link was sent via email to potential respondents with an instruction to respond within two weeks. We aimed to reach a target of 1000, however, because we had to send the survey link to contact persons within organizations and in some states, it is difficult to ascertain the total number of people that was eventually reached. Subsequently, the research team sent an email as a reminder to the potential respondents two weeks after the initial contact. The survey was conducted between September 2018 and January 2019.

Potential participants who had difficulty in participating online had the option of a face-to-face interview or telephone interviews. These interviews were conducted by research assistants who had at least a Bachelor's degree. All the survey data were uploaded by a research assistant on the Qualtrics platform.

Data analysis

Data were first exported from the Qualtrics platform into SPSS statistical software package, version 20. Data were then cleaned and frequencies were used to summarize the demographic characteristics of respondents. . Domain analysis of the CFIR was conducted to highest occurring factor that contributes/and impedes successful community engagement. This was further broken down into internal and external domains contributors. While for barriers to community engagement CFIR domain analysis was conducted to identify most significant domain and further analysis was conducted on construct level to gain more insight on these barriers.

Ethical considerations

For peer review only - http://bmjopen.bmj.com/site/about/guidelines.xhtml

Ethical approval for this study was obtained from the National Health research Ethics Committee (NHREC/01/01/2007). Verbal informed consent was obtained from the study participants who participated in the face to face interviews and telephone interviews verbally after the objectives and rationale of the study were explained. Participants who provided their responses via the Qualtrics platform signed their consent after a detailed description of the study and its rationale was provided. Participants were informed that their responses were anonymous unless they indicated that they would like to be contacted for follow-up and dissemination of survey results. They were assured that their information will remain confidential and will not be shared beyond the research team without their consent

Patient and public involvement

Patients and/or the public were not involved in the design, or conduct, or reporting, or dissemination plans of this research.

Results

A total of 364 of the 966 respondents (37.7%) indicated that they had participated in community engagement activities while working with the polio eradication program. Of these, 249 (68.4%) participants had been engaged in the Nigerian PEI for less than 10 years. The respondents had been engaged in the various levels of implementation of PEI activities; the highest proportion of respondents (57.4%) worked at the local government level. The most common roles included team supervisors (46.9%) and front-line health workers 30.8%. Additional socio-demographic characteristics of survey participants are presented in Table 2. The most common roles included team supervisors (46.9%) and frontline health workers (30.8%). Additional socio-demographic characteristics of survey participants are presented in Table 2.

[Table 2: Socio-demographics characteristics of respondents]

For peer review only - http://bmjopen.bmj.com/site/about/guidelines.xhtml

1: Factors that contributed to the successful implementation of community engagement activities in the PEI, Nigeria

A total of 154 (45.0%) of the participants, reported that the process of conducting the PEI program (for instance, planning of stakeholder engagement, engagement of appropriate stakeholder, executing the activities as planned and monitoring engagement outcomes against the stated objectives) was the most important internal contributor to implementing community engagement activities in the community. This was followed by other internal factors 84 (24.0%) including characteristics of individuals within the organization involved in the PEI activities, awareness of benefits of community engagement, health workers enthusiasm and sustained support throughout the stages of the engagement and commitment to the organization and PEI program characteristics 73 (21.0%) including the positive perception of quality and effectiveness of community engagement activities, the adaptability of the strategy to local context, ease of implementation of the strategy, and minimal challenges encountered during implementation (see Figure 1).

[Figure 1]

The most frequently mentioned external contributor to the community engagement activities was the social environment, 186 (56.0%), in which the community engagement activity was implemented (particularly sociocultural beliefs around immunization). Also, the political factors (stakeholders' and political support) within the communities (88; 26.0%) was a commonly mentioned external contributor. The economic factors, 55 (16.0%), were also mentioned as contributors to community engagement activities (Figure 2).

[Figure 2]

2: Factors that challenge the implementation of community engagement activities in the PEI, Nigeria

For peer review only - http://bmjopen.bmj.com/site/about/guidelines.xhtml

The external environment, 182(50%) (for example, stakeholders' lack of interest poor funding mechanisms and norms that hinder immunization campaigns) was the most implicated factor in the challenges to community engagement activities followed by process of conducting the community engagement activities, 84(23.1%), and the characteristics of people involved in the polio eradication activities, 82(22.5%) (e.g. lack of awareness of benefits of the vaccine, poor knowledge of principles of vaccination (see Figure 3).

[Figure 3]

The respondents indicated that within the external environment domain, the economic environment 65(35.7%) (e.g. funding mechanism in place) and the social environment, 60(32.9%) (e.g. sociocultural beliefs about vaccination), were the most frequent external challenges to community engagement. Other challenges highlighted were the political environment 49(26.9%) such as, political will and set priorities, and the technological environment, 17(9.3%) (including access to technology). The global climate and ineffective cross-organizational collaborations were mentioned 53(29.1%) as challenging factors in the category of other environments.

As shown in Table 3, of all the processes involved in conducting PEI program activities, the process of conducting community engagement (for example, types of activities and stakeholders involved and how the activities were organized), 39(46.4%) was the most important challenge to implementing community engagement strategies. This was closely followed by the execution phase, 37(44.0%), and then the planning phase, 30(35.7%), and the reflection phase, 23(27.4%).

In the domain exploring the characteristics of individuals involved in the PEI activities within the organization, the individual's knowledge about the importance of community engagement and belief that community engagement may not be effective was the mostly reported challenge (62.2%) in terms of effectively engaging community members during implementation of PEI activities. Other factors identified in this category were the individual's perception about and degree of commitment to the PEI, 23(28.0%), the individual's stage of change 22(26.8%), self-efficacy 11(13.4%) and other personal attributes 4(4.9%). See details in Table 3.

For peer review only - http://bmjopen.bmj.com/site/about/guidelines.xhtml

According to respondents, the level of readiness for implementation within the PEI organization contributed the most 28(46.7%) to successfully engaging the community in PEI activities. This was closely followed by the level of networking and communication within the organization 21(35.0%). The culture, implementation climate and structural characteristics within the PEI organization contributed the least to community engagement at 17(28.3%), 16(26.7%) and 11(18.3%) respectively (Table 3).

[Table 3: Categories of factors that impede the implementation of community engagement activities]

The PEI program factors which contributed to effective community engagement were intervention source, 17(37.8%), and adaptability of the program to the local context, 17(37.8%). The cost of the program was also noted to be of relative significant importance to community engagement 14(31.1%). Evidence strength and relative advantage were reported to equally contribute to community engagement, 12(26.7%) each. The complexity, design quality and trialability of the program least contributed to community engagement at 10(22.2%), 9(20.0%) and 5(11.1%), respectively.

Discussion

This paper identified the process of conducting community engagement activities (such as planning of stakeholder engagement, engagement of appropriate stakeholder, executing the activities as planned and monitoring engagement outcomes against intended objectives) as a major contributor to the success of community engagement strategy under the PEI program in Nigeria. Characteristics of individuals (for instance awareness of benefits of the community engagement, health workers enthusiastic and sustained support throughout the stages of the community engagement and commitment to the organization) implementing the activities were

For peer review only - http://bmjopen.bmj.com/site/about/guidelines.xhtml

identified as another contributor, while the external environment, especially the economic and social environment (for example, sociocultural beliefs that hinder immunization such as family socio economic status, mothers literacy level, husbands permission before immunization, religious beliefs about vaccines, rumors and misconception surrounding vaccines, side effects of vaccination), served as a major barrier to community engagement. Hence, when properly harnessed, the social environment can contribute to the success of the community engagement goals but if neglected can hinder the successful implementation of a community engagement strategy, just like many authors have reported.[28, 29]

In order to harness these factors (process of conducting activities, individual characteristics, socio and economic environment), it is necessary to think about how readily modifiable these factors are. The process of conducting community engagement is a readily modifiable factor as it has evolved to a strategy that is led by the community members to ensure sustainability and ownership of the engagement process. While the characteristics of the PEI workforce have been modified over the years by equipping them with skills and abilities to provide sustained support to community engagement and even changing the gender composition of members to address social beliefs.[20] However, the social cultural realities of a community are not readily modifiable because this is the way of life of the community. The PEI has successfully highlighted aspects of the culture that supports vaccination and addresses myths, beliefs and misconceptions that hinder participating in the immunization exercises.[30] Therefore, finding a balance between the positives and negatives of any community's culture should be the goal of community engagement activities and not modifying the community's culture.

In the *internal setting,* there is an interplay of vital factors such as the process of conducting activities and characteristics of individuals within the organization. According to Damschroder and colleagues, essential components for the success of community engagement activities include the process of conducting activities that involved the planning of the activities, the mode of engagement and the point of entry into the community.[19] An instance of this interplay is an unmotivated health worker given a poorly planned program to implement, this increases the possibility of failure in implementation failure. Other essential considerations for successful community engagement were: the characteristics of individuals to involve and how the community engagement is executed, monitored and evaluated.[19] The polio program has

For peer review only - http://bmjopen.bmj.com/site/about/guidelines.xhtml

documented the importance of measuring staff performance for the accountability framework and how it plays a role in achieving effective acute flaccid paralysis (AFP) surveillance. Therefore, it is believed that harnessing staff performance which falls in the category of characteristics of individuals involved for community engagement will show similar positive results.[31]

Based on the individuals involved in the program reflections on Africa's polio campaigns shows that any initiative is only as good as the quality of the human resource engaging with the community.[32] The PEI focused on local champions called the volunteer community mobilizers who advocate and align with the goal of the program. They were purposeful in addressing community challenges (non-compliant households, vaccine-rejection cases, missed children) in Northern Nigeria.[33] Engagement of these individuals in the polio programs, addressing their misconceptions about the benefits of the polio program and improving their self-efficacy to share information on polio immunization has increased a sense of ownership of the polio program which was the primary goal of community engagement, encouraged participation of religious and traditional leaders in the program and developed social networks within the community will be available to work on routine immunization and other health services as part of the polio legacy.[6]

The *external setting,* the social environment was identified as the most influential factor in supporting as well as undermining the community engagement process. In line with the submissions of several authors,[28, 29] the unique context, culture, beliefs, modes of communication of any community could be a threat or facilitator of community participation in any program. In the case of the polio program in the northern part of Nigeria, the culture of preventing visitors contact with their wives, beliefs about the content of the polio vaccines, their nomadic way of life and their social norms about immunization activities negatively affected the coverage rates in these areas.[13, 34, 35] However, engagement of the community members resulted in identification of the problems and proffering of mutually acceptable solutions.[36]

This resistance to immunization due to misconceptions and cultural beliefs in northern Nigeria was not as pronounced in the other parts of the country.[13, 34] This shows a sharp contrast between the northern and the southern regions of the country.[13] In essence, this shows that for health interventions to be successful there should be some measure of contextual basis to it

For peer review only - http://bmjopen.bmj.com/site/about/guidelines.xhtml

implementation. The socio-cultural context of communities should be considered and extensive dialogue carried out to ensure communities are adequately represented and take ownership of the program. Similar to these, findings from previous studies on implementation research have also emphasized the importance of an intervention being contextual and flexible enough to be tailored to fit different environments.[27, 37]

This study also highlighted another external factor, the political environment of the community. The PEI program in Nigeria has over the years emphasized the importance of the political context in the success of any strategy both at the national scale and down to the grass-root level. For the PEI to succeed, it was important to win over political factions to increase the political will of the community representatives, avoid political interference in its activities so to make sure all community members benefit from the program regardless of political factions.[30, 38] Therefore, in addition to understanding the social context of the community, it is necessary that any community engagement seeks to understand the politics within the community, the various political factions with their beliefs and grievances. Most importantly, however, implementers must strive to remain politically neutral, to ensure that no section of the community is unrepresented due to their political affiliations.

The economic environment which involved the availability of sufficient revenues to fund immunization activities and the primary health care system were the most influential external environmental factor considered by implementers of community engagement activities. For the polio program, the Interagency Coordinating Committee (ICC) and other GPEI partners responsible for providing considerable funds to the program. These funds were used to set up 16 fully-equipped laboratories to process stool and environmental samples both for the polio program and other vaccine-preventable diseases and support the primary health system to deliver other maternal and child health interventions. The provision of health infrastructures is one of the legacies of the PEI.[39] Consequently, there have been adequate funds for the successful implementation of the polio program and the support community engagement in various communities for the success of their agenda. This factor shows that engaging community members goes beyond understanding the context of the community or just advocating for increased political will. It also involves funding to recruit community members as volunteers, to provide stipends or provide logistics support to religious and traditional leaders when they attend

For peer review only - http://bmjopen.bmj.com/site/about/guidelines.xhtml

dialogue meeting or even providing incentives to caregivers. Therefore, future program implementers should budget for the funding for community engagement activities and the sustainability of those funding for these activities even after the achievement of the project goals. This finding is corroborated by Andrus who highlighted the role of the funds in success of programs by stating that a constant demotivator for polio personnel across endemic countries has constantly been the lack of financial incentives.[33] While Bigna[24] summarized it in his words, "*Money is the nerve of war, so, going to war against wild poliovirus will require sufficient funding to destroy it*".

Study limitations

This study utilized a cross-sectional design therefore it was impossible to determine causal relationships between variables. Furthermore, there was a tendency to recall bias as the PEI spanned a few decades. Similarly, social desirability bias was a possibility because participants were involved in the program and would probably not be inclined to present the activities in a negative light. These limitations notwithstanding, this structured assessment provided useful data that policy makers and program managers may use to promote community engagement and gain community trust thereby ensuring successful community engagement.

Conclusion

The CFIR revealed specific contextual factors that influence effectiveness of community engagement implementation. The *process of conducting the activities and the external environment* (economic, social and political) were very critical from this study. Program implementers should collaborate transparently with the community, foster trust and maintain a relationship with the community. These findings contribute to implementation research literature supporting the use of the CFIR in displaying constructs and domains that influence health innovation implementation. These findings can be used by policymakers and researchers to advocate for effective ways to implement community engagement activities.

Acknowledgments

We appreciate all the participants in this study. We are also grateful to members of the STRIPE consortium led by the Johns Hopkins University Bloomberg School of Public Health for their contributions at different times to the STRIPE protocol.

For peer review only - http://bmjopen.bmj.com/site/about/guidelines.xhtml

Authors' contributions

This manuscript was conceived by ETO, AA and OA. The initial draft was developed by AA, CB and CN and reviewed by OA, AF, AK, OOA and ETO. The final version of the manuscript was approved by OA, AA, CB, CN, AK, AF, OOA and ETO.

Provenance and peer review

Not commissioned; externally peer reviewed

Availability of data and materials

The data used and/or analysed in this study are available from the corresponding author on request.

Disclaimer

The funders did not have any role in study design, data collection and analysis, decision to publish, or preparation of the manuscript.

Competing interests: None declared.

Patient and public involvement: Patients and/or the public were not involved in the design, or conduct, or reporting, or dissemination plans of this research.

Patient consent for publication

Not applicable.

Abbreviations

**CFIR:**	Consolidated Framework for Implementation Research
**CGPP:**	CORE Group Partners Project
**GPEI:**	Global Polio Eradication Initiative
**NPHCDA:**	National Primary Healthcare Development Agency
**PEI:**	Polio Eradication Initiative
**STRIPE:**	Synthesis and Translation of Research and Innovations from Polio Eradication
**VCM:**	Volunteer Community Mobilizers

For peer review only - http://bmjopen.bmj.com/site/about/guidelines.xhtml

References

1. Callaghan KA. Establishing Community-Based Primary Health Care. Community-Based Health Interventions in an Institutional Context: Springer; 2019. p. 23-38.

2. Alves OMA, Moreira JP, Santos PC. Developing community partnerships for primary healthcare: An integrative review on management challenges. International Journal of Healthcare Management. 2020:1-19.

3. Hammanyero KI, Bawa S, Braka F, Bassey BE, Fatiregun A, Warigon C, et al. Lessons learnt from implementing community engagement interventions in mobile hard-to-reach (HTR) projects in Nigeria, 2014–2015. BMC Public Health. 2018; 18:1306.

4. World Health Organization, Global Polio Eradication Initiative. Polio endgame strategy 2019-2023: eradication, integration, certification and containment: World Health Organization2019.

5. Habib MA, Soofi S, Cousens S, Anwar S, ul Haque N, Ahmed I, et al. Community engagement and integrated health and polio immunisation campaigns in conflict-affected areas of Pakistan: a cluster randomised controlled trial. The Lancet Global Health. 2017; 5:e593-e603.

6. McArthur-Lloyd A, McKenzie A, Findley SE, Green C, Adamu F. Community engagement, routine immunization, and the polio legacy in northern Nigeria. Global Health Communication. 2016; 2:1-10.

7. Nasir S-G, Aliyu G, Ya'u I, Gadanya M, Mohammad M, Zubair M, et al. From intense rejection to advocacy: How Muslim clerics were engaged in a polio eradication initiative in Northern Nigeria. PLoS Med. 2014; 11:e1001687.

8. Fekadu L, Okeibunor J, Nsubuga P, Kipela JM, Mkanda P, Mihigo R. Reaching the unreached with polio vaccine and other child survival interventions through partnership with military in Angola. Vaccine. 2016; 34:5155-8.

9. Ongwae KM, Bawa SB, Shuaib F, Braka F, Corkum M, Isa HK. Use of Dedicated mobile teams and polio volunteer community mobilizers to increase access to zero-dose oral poliovirus vaccine and routine childhood immunizations in settlements at high risk for polio transmission in northern Nigeria. The Journal of infectious diseases. 2017; 216:S267-S72.

10. Setswe G, Witthuhn J. Community engagement in the introduction and implementation of the National Health Insurance in South Africa. Journal of Public Health in Africa. 2013; 4.

For peer review only - http://bmjopen.bmj.com/site/about/guidelines.xhtml

11. Dalhatu S, Ghani NA, Bozkurt V. A systematic review on factors affecting community participation towards polio immunization in Nigeria. Mediterranean Journal of Social Sciences. 2015; 6:407-.

12. Van Den Ent MM, Swift RD, Anaokar S, Hegg LA, Eggers R, Cochi SL. Contribution of global polio eradication initiative–funded personnel to the strengthening of routine immunization programs in the 10 focus countries of the polio eradication and endgame strategic plan. The Journal of Infectious Diseases. 2017; 216:S244-S9.

13. Ghinai I, Willott C, Dadari I, Larson HJ. Listening to the rumours: What the northern Nigeria polio vaccine boycott can tell us ten years on. Global public health. 2013; 8:1138-50.

14. Jegede AS. What led to the Nigerian boycott of the polio vaccination campaign? PLoS Med. 2007; 4:e73.

15. Cochi SL, Freeman A, Guirguis S, Jafari H, Aylward B. Global polio eradication initiative: lessons learned and legacy. The Journal of infectious diseases. 2014; 210:S540-S6.

16. Cochi SL, Hegg L, Kaur A, Pandak C, Jafari H. The global polio eradication initiative: progress, lessons learned, and polio legacy transition planning. Health Affairs. 2016; 35:277-83.

17. Aylward RB, Acharya A, England S, Agocs M, Linkins J. Global health goals: lessons from the worldwide effort to eradicate poliomyelitis. The Lancet. 2003; 362:909-14.

18. Nasir UN, Bandyopadhyay AS, Montagnani F, Akite JE, Mungu EB, Uche IV, et al. Polio elimination in Nigeria: A review. Human Vaccines & Immunotherapeutics. 2016; 12:658-63.

19. Damschroder LJ, Aron DC, Keith RE, Kirsh SR, Alexander JA, Lowery JC. Fostering implementation of health services research findings into practice: a consolidated framework for advancing implementation science. Implementation science. 2009; 4:1-15.

20. Alonge O, Neel A, Kalbarczyk A, Peters M, Mahendradhata Y, Sarker M, et al. Synthesis and translation of research and innovations from polio eradication (STRIPE): Initial findings from a global mixed methods study. BMC Public Health. 2020; 20.

21. CIA. The World Factbook. Central Intelligence Agency. 2012.

22. Renne E. Perspectives on polio and immunization in Northern Nigeria. Social science & medicine. 2006; 63:1857-69.

23. Hamisu AW, Johnson TM, Craig K, Mkanda P, Banda R, Tegegne SG, et al. Strategies for improving polio surveillance performance in the security-challenged Nigerian States of

For peer review only - http://bmjopen.bmj.com/site/about/guidelines.xhtml

Adamawa, Borno, and Yobe during 2009–2014. The Journal of infectious diseases. 2016; 213:S136-S9.

24. Bigna JJR. Polio eradication efforts in regions of geopolitical strife: the Boko Haram threat to efforts in sub-Saharan Africa. African Health Sciences. 2016; 16:584-7.

25. Abimbola S, Malik AU, Mansoor GF. The final push for polio eradication: addressing the challenge of violence in Afghanistan, Pakistan, and Nigeria. PLoS Med. 2013; 10:e1001529.

26. NAN. WHO declares Nigeria Polio free. The Guardian. 2020 19 June 2020.

27. Saluja S, Silverstein A, Mukhopadhyay S, Lin Y, Raykar N, Keshavjee S, et al. Using the Consolidated Framework for Implementation Research to implement and evaluate national surgical planning. BMJ global health. 2017; 2.

28. Pell CL, Adhikari B, Myo Thwin M, Kajeechiwa L, Nosten S, Nosten FH, et al. Community engagement, social context and coverage of mass anti-malarial administration: Comparative findings from multi-site research in the Greater Mekong sub-Region. PLoS One. 2019; 14:e0214280.

29. Sahan K, Pell C, Smithuis F, Phyo AK, Maung SM, Indrasuta C, et al. Community engagement and the social context of targeted malaria treatment: a qualitative study in Kayin (Karen) State, Myanmar. Malaria journal. 2017; 16:1-10.

30. Usman S, Bologna L, Stamidis KV. The CORE Group Partners Project in North East Nigeria: community engagement strategies to combat skepticism and build trust for vaccine acceptance. The American journal of tropical medicine and hygiene. 2019; 101:68-73.

31. Kassahun A, Braka F, Gallagher K, Gebriel AW, Nsubuga P, M'pele-Kilebou P. Introducing an accountability framework for polio eradication in Ethiopia: results from the first year of implementation 2014-2015. The Pan African Medical Journal. 2017; 27.

32. Kumar P, Khan A. Human resource management in primary health care system. Health and Population–Perspectives and Issues. 2013; 36:66-76.

33. Andrus JK, Perry HB. Community engagement, ownership, and civil society organizations in polio eradication. The American journal of tropical medicine and hygiene. 2019; 101:1.

34. Yahya M. Polio vaccines—"no thank you!" barriers to polio eradication in Northern Nigeria. African Affairs. 2007; 106:185-204.

For peer review only - http://bmjopen.bmj.com/site/about/guidelines.xhtml

35. Taylor SA. Culture and behaviour in mass health interventions: lessons from the global polio eradication initiative. Critical Public Health. 2015; 25:192-204.

36. Coates EA, Waisbord S, Awale J, Solomon R, Dey R. Successful polio eradication in Uttar Pradesh, India: the pivotal contribution of the Social Mobilization Network, an NGO/UNICEF collaboration. Global Health: Science and Practice. 2013; 1:68-83.

37. Akinyemi O, Harris B, Kawonga M. Health system readiness for innovation scale-up: the experience of community-based distribution of injectable contraceptives in Nigeria. BMC health services research. 2019; 19:1-11.

38. Haq Z, Shaikh BT, Tran N, Hafeez A, Ghaffar A. System within systems: challenges and opportunities for the Expanded Programme on Immunisation in Pakistan. Health research policy and systems. 2019; 17:51.

39. Craig AS, Haydarov R, O'Malley H, Galway M, Dao H, Ngongo N, et al. The public health legacy of polio eradication in Africa. The Journal of Infectious Diseases. 2017; 216:S343-S50.

For peer review only

Tables

Table 1: CFIR domains and associated constructs

Construct		Short Description
**Internal setting**		
**I. Intervention/PEI program characteristics**		
A	Intervention Source	Perception of key stakeholders about whether the intervention is externally or internally developed.
B	Evidence Strength & Quality	Stakeholders' perceptions of the quality and validity of evidence supporting the belief that the intervention will have desired outcomes.
C	Relative Advantage	Stakeholders' perception of the advantage of implementing the intervention versus an alternative solution.
D	Adaptability	The degree to which an intervention can be adapted, tailored, refined, or reinvented to meet local needs.
E	Trialability	The ability to test the intervention on a small scale in the organization, and to be able to reverse course (undo implementation) if warranted.
F	Complexity	Perceived difficulty of implementation, reflected by duration, scope, radicalness, disruptiveness, centrality and intricacy and number of steps required to implement.
G	Design Quality & Packaging	Perceived excellence in how the intervention was bundled, presented, and assembled.
H	Cost	Costs of the intervention and costs associated with implementing the intervention including investment, supply, and opportunity costs.
**II. Inner/Organizational settings**		
A	Structural Characteristics	The social architecture, age, maturity, and size of an organization.
B	Networks & Communications	The nature and quality of webs of social networks and the nature and quality of formal and informal communications within an organization.
C	Culture	Norms, values, and basic assumptions of a given organization.
D	Implementation Climate	The absorptive capacity for change, shared receptivity of involved individuals to an intervention, and the extent to which use of that intervention will be rewarded, supported, and expected within their organization.
E	Readiness for Implementation	Tangible and immediate indicators of organizational commitment to its decision to implement an intervention – leadership engagement and resources available.
F	Others	Other personal attributes
**III. Characteristics of individuals within your organization involved in PEI activities**		
A	Knowledge & Beliefs about the Intervention	Individuals' attitudes toward and value placed on the intervention as well as familiarity with facts, truths, and principles related to the intervention.

For peer review only - http://bmjopen.bmj.com/site/about/guidelines.xhtml

COMMUNITY ENGAGEMENT IN NIGERIA POLIO ERADICATION

B	Self-efficacy	Individual belief in their capabilities to execute courses of action to achieve implementation goals.
C	Individual Stage of Change	Characterization of the phase an individual is in, as he or she progresses toward skilled, enthusiastic, and sustained use of the intervention.
D	Individual Identification with Organization	A broad construct related to how individuals perceive the organization, and their relationship and degree of commitment with that organization.
E	Other Personal Attributes	A broad construct to include other personal traits such as tolerance of ambiguity, intellectual ability, motivation, values, competence, capacity, and learning style.
**IV. Process of conducting the activities**		
A	Planning	The degree to which a scheme or method of behavior and tasks for implementing an intervention are developed in advance, and the quality of those schemes or methods.
B	Engaging	Attracting and involving appropriate individuals in the implementation and use of the intervention through a combined strategy of social marketing, education, role modeling, training, and other similar activities.
C	Executing	Carrying out or accomplishing the implementation according to plan.
D	Reflecting & Evaluating	Quantitative and qualitative feedback about the progress and quality of implementation accompanied with regular personal and team debriefing about progress and experience.
**V. External/Outer setting**		
A	Political environment	Lawmaker support, political climate accepting of polio eradication activities, and political structure to conducive to coordinated action
B	Economic environment	Sufficient revenue sources/base to fund activities and/or maintain system developments
C	Social environment	Social norms around immunization, accepting communities in which polio eradication activities were implemented
D	Technological environment	Infrastructure or technological advances outside of the organization
E	Other environment	Environment where activity was implemented was prohibitive and did not contribute to the success of polio eradication, including the global climate and ineffective cross-organizational collaboration

Adapted from a study [16] on Fostering implementation of health services research findings into practice: A Consolidated Framework for advancing Implementations Science.

For peer review only - http://bmjopen.bmj.com/site/about/guidelines.xhtml

Table 2: Socio-demographics characteristics of respondents

Respondents characteristics	n(%)
**Work experience (years)**	
<10	249(68.4)
10-19	96(26.4)
≥20	19(5.2)
**Level of involvement***	
Global	21(5.8)
National	64(17.6)
State	168(46.2)
District	209(57.4)
Sub district	120(32.9)
**Affiliated organization***	
Government agencies	348(96.6)
Other GPEI Partners	234(64.3)
UNICEF	147(40.4)
Implementing partners	163(44.8)
Global NGO	20(5.5)
Academic research	5(1.4)
**Roles with the PEI program in Nigeria***	
Team supervisor	171(46.9)
Frontline health worker	112(30.8)
Others**	101(27.7)
Monitoring and oversight board	78(21.4)
Strategy committee/management group	57(15.6)
Program officer	37(10.2)
Surveillance officer	35(9.6)
EPI manager	32(8.8)
Program manager	25(6.9)
Policymaker	12(3.3)
Researcher	7(1.9)
Country project lead	6(1.6)

**Multiple response questions; **Others include: Consultant, Cluster Coordinator, Data Analysis Officer, Medical Officer of Health*

For peer review only

For peer review only - http://bmjopen.bmj.com/site/about/guidelines.xhtml

Table 3: Categories of factors that impede the implementation of community engagement activities*

Factors	Categories	n(%)
External environment	Economic	65(35.7)
	Social	60(32.9)
	Political	49(26.9)
	Technological	17(9.3)
	Other environments	53(29.1)
Processes involved in conducting PEI program activities	Engagement phase	39(46.4)
	Execution phase	37(44.0)
	Planning phase	30(35.7)
	Reflection phase	23(27.4)
Intervention/PEI program characteristics	Intervention source	17(37.8)
	Adaptability of the program to local context	17(37.8)
	Cost	14(31.1)
	Evidence strength and	12(26.7)
	Relative advantage	12(26.7)
	Complexity	10(22.2)
	Design quality	9(20.0)
	Trialability	5(11.1)
Inner/Organizational settings	Level of readiness for implementation	28(46.7)
	Networks and Communication	21(35.0)
	Culture	17(28.3)
	Implementation of climate	16(26.7)
	Structural characteristics	11(18.3)

For peer review only

Characteristics of individuals involved within the organization	Individual's knowledge and beliefs about the activity	51(62.2)
	Individual's identification with the organization	23(28.0)
	The individual's stage of change	22(26.8)
	Self-efficacy	11(13.4)
	Other personal attributes	4(4.9)

******Multiple response*

For peer review only

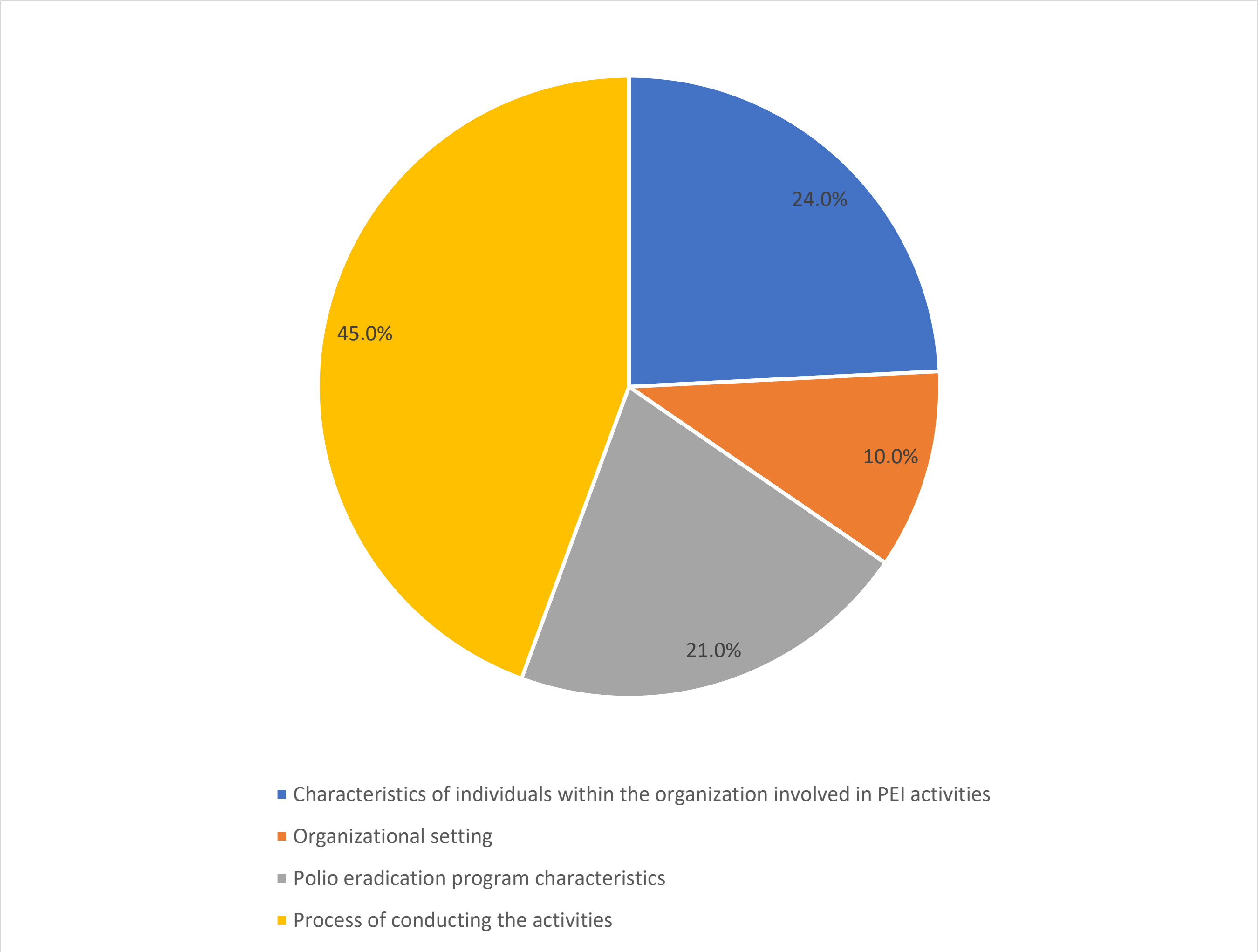

Figure 1: Internal contributors to the successful implementation of community engagement activities.

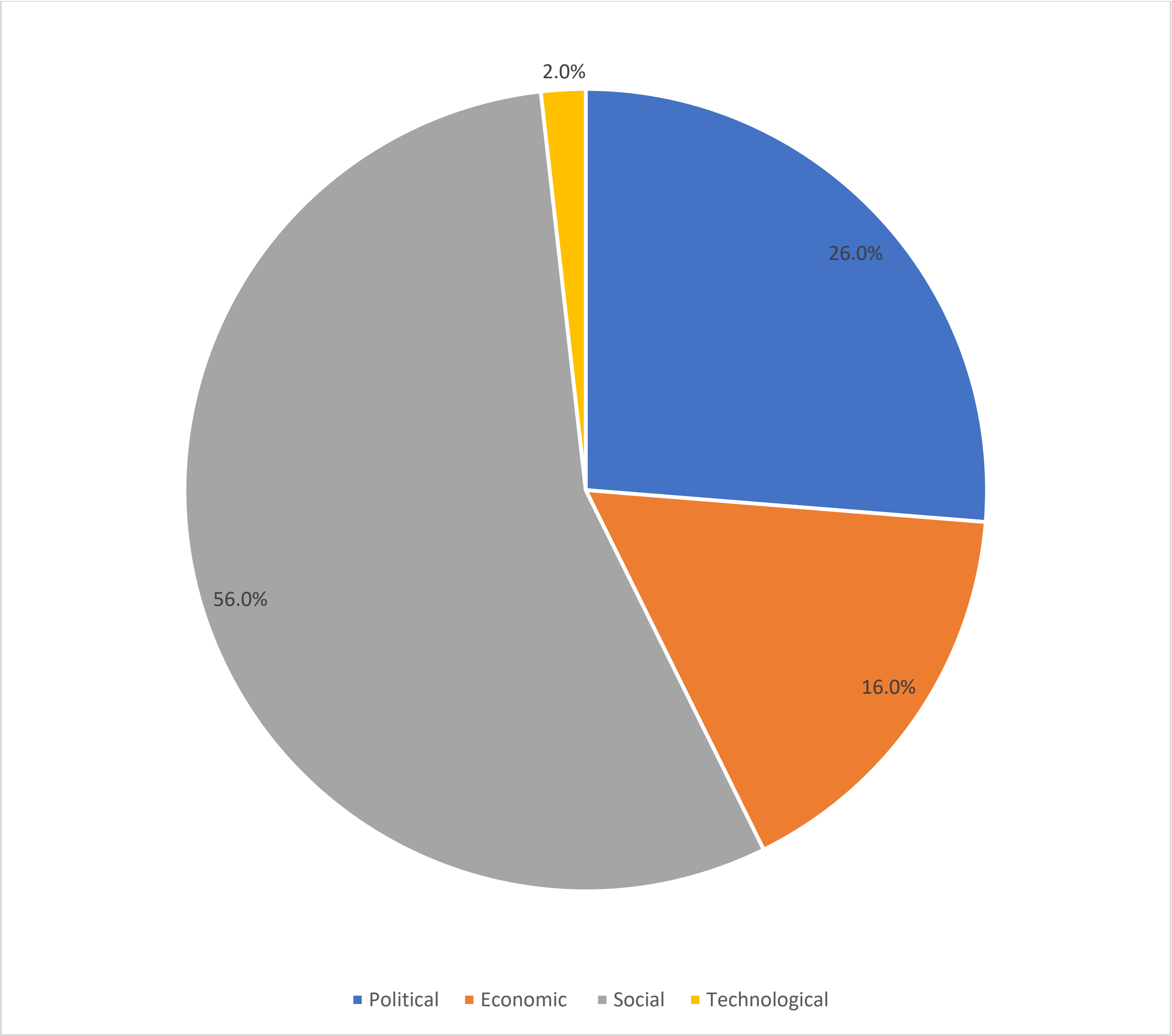

Figure 2: External contributors to the successful implementation of community engagement activities.

For peer review only

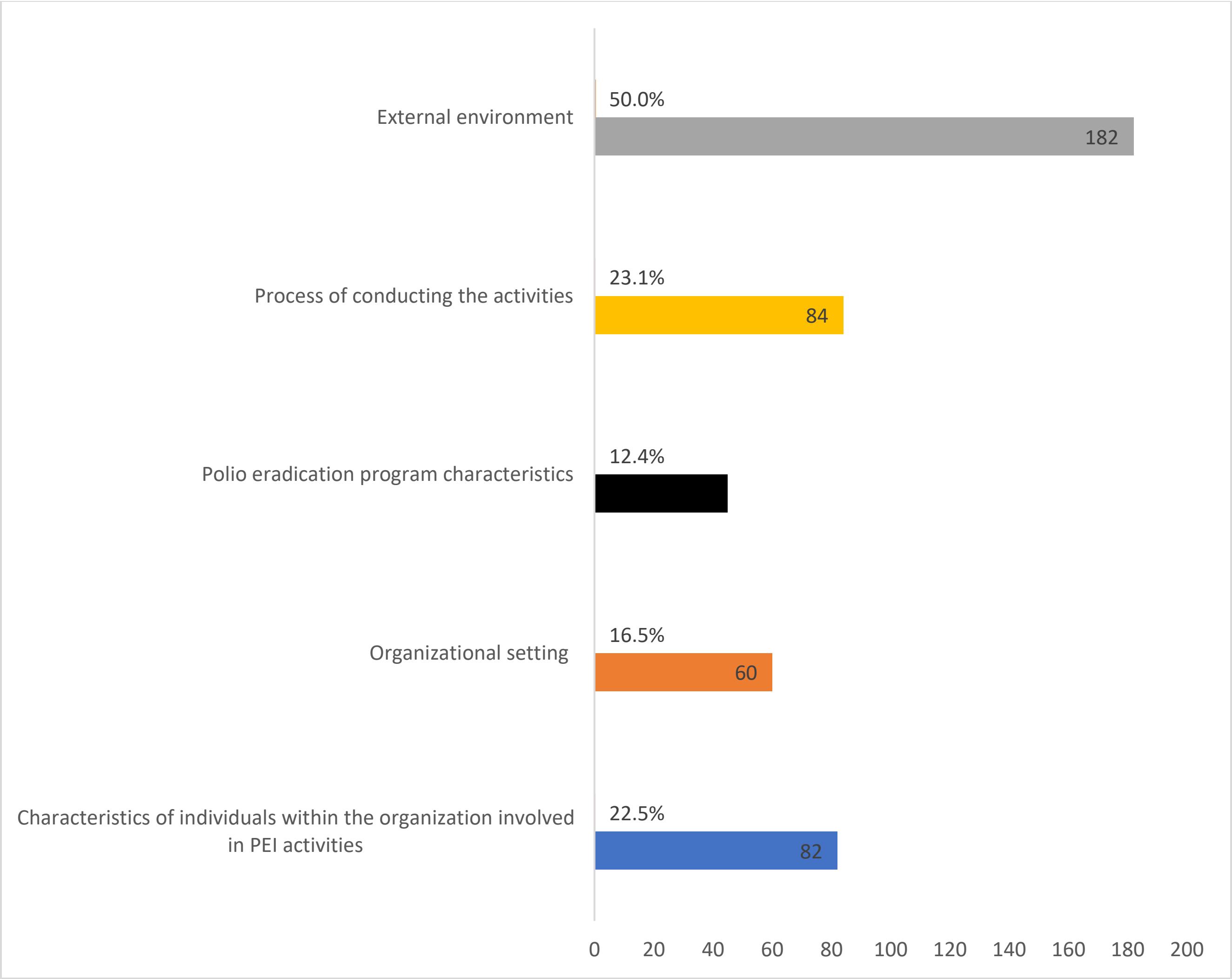

Figure 3: Factors that impede the implementation of community engagement activities.

BMJ Open

Assessing community engagement in Nigeria polio eradication initiative: application of the Consolidated Framework for Implementation Research

Journal:	*BMJ Open*
Manuscript ID	bmjopen-2021-048694.R1
Article Type:	Original research
Date Submitted by the Author:	22-Jun-2021
Complete List of Authors:	Akinyemi, Oluwaseun; University of Ibadan College of Medicine, Health Policy and Management<br>Adebayo, Adedamola; University of Ibadan College of Medicine, Department of Community Medicine<br>Bassey, Christopher ; University of Ibadan College of Medicine, Department of Community Medicine<br>Nwaiwu, Chioma ; University of Ibadan College of Medicine, Department of Community Medicine<br>Kalbarczyk, Anna; Johns Hopkins University Bloomberg School of Public Health, International Health<br>Fatiregun, Akinola; World Health Organization Country Office for Nigeria, Ondo State Field Office<br>Alonge, Olakunle; Johns Hopkins University Bloomberg School of Public Health,<br>Owoaje, Eme; University of Ibadan College of Medicine, Community Medicine
**Primary Subject Heading**:	Public health
Secondary Subject Heading:	Public health, Health policy, Health services research
Keywords:	Public health < INFECTIOUS DISEASES, Health policy < HEALTH SERVICES ADMINISTRATION & MANAGEMENT, Paediatric infectious disease & immunisation < PAEDIATRICS

SCHOLARONE™ Manuscripts

I, the Submitting Author has the right to grant and does grant on behalf of all authors of the Work (as defined in the below author licence), an exclusive licence and/or a non-exclusive licence for contributions from authors who are: i) UK Crown employees; ii) where BMJ has agreed a CC-BY licence shall apply, and/or iii) in accordance with the terms applicable for US Federal Government officers or employees acting as part of their official duties; on a worldwide, perpetual, irrevocable, royalty-free basis to BMJ Publishing Group Ltd ("BMJ") its licensees and where the relevant Journal is co-owned by BMJ to the co-owners of the Journal, to publish the Work in this journal and any other BMJ products and to exploit all rights, as set out in our licence.

The Submitting Author accepts and understands that any supply made under these terms is made by BMJ to the Submitting Author unless you are acting as an employee on behalf of your employer or a postgraduate student of an affiliated institution which is paying any applicable article publishing charge ("APC") for Open Access articles. Where the Submitting Author wishes to make the Work available on an Open Access basis (and intends to pay the relevant APC), the terms of reuse of such Open Access shall be governed by a Creative Commons licence – details of these licences and which Creative Commons licence will apply to this Work are set out in our licence referred to above.

Other than as permitted in any relevant BMJ Author's Self Archiving Policies, I confirm this Work has not been accepted for publication elsewhere, is not being considered for publication elsewhere and does not duplicate material already published. I confirm all authors consent to publication of this Work and authorise the granting of this licence.

For peer review only

Assessing community engagement in Nigeria polio eradication initiative: application of the Consolidated Framework for Implementation Research

Oluwaseun Oladapo Akinyemi,[a*] (ooakinyemi@comui.edu.ng,seunakinyemi@hotmail.com)
Adedamola Adebayo,[b] (adewaledamola@gmail.com)
Christopher Bassey,[b] (christopher.bassey@gmail.com)
Chioma Nwaiwu,[b] (chiomanwaiwu71@yahoo.com)
Anna Kalbarczyk,[c] (akalbarc@jhu.edu)
Akinola A. Fatiregun,[d] (akinfati@yahoo.com)
Olakunle Alonge, [c] (oalonge1@jhu.edu)
Eme T. Owoaje, [b] (emetheodora@gmail.com)

[a] *Department of Health Policy and Management, College of Medicine, University of Ibadan, Ibadan, Nigeria*

[b] *Department of Community Medicine, College of Medicine, University of Ibadan, Ibadan, Nigeria*

[c] *Johns Hopkins Bloomberg School of Public Health, USA*

[d] *World Health Organization, Country Office for Nigeria, Ondo State Field Office, Ondo, Nigeria*

Original research article

***Corresponding author:** Dr Oluwaseun O. Akinyemi
Department of Health Policy and Management,
College of Medicine, University of Ibadan, Ibadan, Nigeria
+234 803 502 0136

Keywords: Community engagement, Polio eradication initiative, Consolidated framework for implementation research, Implementation science

Word counts:

Abstract: 294

Manuscript: 4942

For peer review only - http://bmjopen.bmj.com/site/about/guidelines.xhtml

Assessing community engagement in Nigeria polio eradication initiative: application of the Consolidated Framework for Implementation Research

Abstract

Objective: This study employed the Consolidated Framework for Implementation Research (CFIR) to assess factors that enhanced or impeded the implementation of community engagement strategies using the Nigerian polio program as a point of reference.

Design: This study was a part of a larger descriptive cross-sectional survey. The CFIR was utilized to design the instrument which was administered through face to face and phone interviews as well as a web-based data collection platform, Qualtrics.

Setting: The study took place in at least one State from each of the six geopolitical zones in Nigeria (Nasarawa, Borno, Kano, Sokoto, Anambra, Bayelsa, Lagos, Ondo and Oyo States as well as the Federal Capital Territory).

Participants: The respondents included program managers, policymakers, researchers and frontline field implementers affiliated with the Global Polio Eradication Initiative core partner organizations, the three tiers of the government health parastatals (local, state and federal levels) and academic/research institutions.

Results: Data for this study were obtained from 364 respondents who reported participation in community engagement activities in Nigeria's Polio Eradication Initiative (PEI). Majority (68.4%) had less than 10 years' experience in PEI, 57.4% were involved at the local government level and 46.9% were team supervisors. Almost half (45.0%) of the participants identified the process of conducting the PEI program and social environment (56.0%) as the most important internal and external contributor to implementing community engagement activities in the community, respectively. The economic environment (35.7%) was the most frequently reported challenge among the external challenges to implementing community engagement activities.

Conclusion: Community engagement strategies were largely affected by factors relating to the process of conducting the polio program, the economic environment and the social context. Therefore, community engagement implementers should focus on these key areas and channel resources to reduce obstacles to achieve community engagement goals.

For peer review only - http://bmjopen.bmj.com/site/about/guidelines.xhtml

Strengths and limitations of this study

- This study presents findings of one of the most important strategies of the polio eradication program and presented findings which are useful for public health program implementers in achieving community engagement objectives
- The participants in this study are a representative of all categories of stakeholders from the national to the local levels that participated in the polio program thereby bringing in their nuanced insights.
- Social desirability bias was a possibility because participants were involved in the program and would probably not be inclined to present the activities in a negative light.
- This study utilized a cross-sectional design therefore it was impossible to determine causal relationships between variables. Furthermore, there was a tendency to recall bias as the PEI spanned a few decades.

Introduction

Community engagement is a fundamental principle in the planning, organizing and delivery of primary health care services to the community.[1, 2] This principle, set during the Alma-Ata declaration of 1978, remains relevant in the Sustainable Development Goals (SDG) era when engaging the community is the priority for the achievement of universal health coverage by 2030.[3] The benefits of engaging the community in the planning and implementation phases of health interventions have been documented across various maternal and child health programs, including the polio eradication program.[4-6] In the primary health care system, this strategy supports relationship building with community members,[7] reduces health inequalities,[8] provides a channel for the dissemination of information on health products and services and aids the health system to adapt the health service to the local context and address misinformation about health interventions.[9, 10]

The implementation of community engagement strategies in the polio program has enhanced coverage, acceptability and ownership of health programs in high-risk areas such as hard-to-reach and conflict-affected communities, which would have otherwise been difficult to achieve.[5] Community engagement has been beneficial and has encouraged the adoption of health services by individuals who have negative beliefs and attitudes by engaging them to discuss the basis of their beliefs and encouraging them to proffer solutions to issues they identified.[11] One successful example of community engagement in high-risk areas includes the

For peer review only - http://bmjopen.bmj.com/site/about/guidelines.xhtml

2014 engagement of 11,000 female community-based mobilizers in Nigeria to advocate for vaccine uptake in northern Nigeria.[12] This resulted in over 322,000 newborns referred for routine immunization and over 32,000 malnourished children referred for appropriate care.[12]

Alternatively, when community engagement was not used and these high-risk communities were neglected during planning and implementation of public health interventions, misconceptions about the program emerged leading to noncompliance to the public health directives or outright rejection of health interventions.[13] The polio program in Nigeria has had first-hand experience with vaccine hesitancy and rejection of the polio vaccines. This was a result of neglecting political and religious leaders' opinions and not addressing their misconceptions before program implementation, especially in Northern Nigeria.[13, 14] This challenge was eventually resolved through dialogue with religious and political leaders and by engaging the community in validating the vaccine.[6] The Global Polio Eradication Initiative (GPEI) then adopted this strategy in addressing other challenges that arose in these communities and in other countries yet to be certified polio-free.[15-17] The community engagement strategy has taken various formats over the years ranging from engaging religious leaders and traditional leaders to engaging opinion leaders and other members of the community. Other community engagement strategies include creating media awareness as well as providing incentives to caregivers and other participating community members. These innovations have yielded substantial benefits to the immunization system and the primary health care system.[4, 18]

The wild poliovirus was last detected in Nigeria in 2016, and community engagement has been identified as a principal strategy for achieving this success.[12] However, as most community engagement activities in polio have been reactionary, there is a gap in our understanding of how to systematically translate best practices in community engagement activities to other programs and contexts. Whereas other Primary Health Care programs have leveraged on the gains of the polio eradication initiative in Nigeria and are adopting the strategies employed by the polio program, including community engagement, the adoption of these strategies has yielded mixed results due to variable implementation – and lack of a systematic model to adapt these strategies to other contexts. Therefore, there is a need to extensively discuss potential factors that could hinder or facilitate the implementation of community engagement strategies using the polio program in Nigeria as a case study.

For peer review only - http://bmjopen.bmj.com/site/about/guidelines.xhtml

The Consolidated Framework for Implementation Research (CFIR),[19] which provides relevant domains and constructs for analyzing public health strategies, can be applied to systematically identify relevant aspects for the implementation of community engagement strategies, without which, such strategies would be unsuccessfully implemented in any context. This study used the CFIR to identify factors that facilitated or hindered the implementation of community engagement activities within the Nigeria's Polio Eradication Initiative (PEI). The study was conducted as part of a multi-country implementation science project, Synthesis and Translation of Research and Innovations from Polio Eradication (STRIPE), to capture lesson learned from the Global Polio Eradication Initiative (GPEI).[20] It is hoped that the knowledge of these factors will enhance the adaptation of community engagement activities in other public health programs, and the effective delivery of these programs in Nigeria and similar settings.

Methods

Study setting

Nigeria is the most populous country in Sub-Saharan Africa with an estimated population of 200 million persons of which over two-thirds are children and young persons.[21] Nigeria is divided into six geopolitical zones namely South-South, South-East, South-West, North-East, North-Central and North-West. Overall, Northwest, Kano-Sokoto hub, have the highest number of cases of poliomyelitis followed by the Northeast, Borno -Yobe hub.[22]

In recent times, Nigeria has experienced security challenges initially from the militants in the south and insurgents and armed bandits in the northern part of the country. This has put health workers engaged in vaccination efforts in jeopardy.[23, 24] Consequently, the country missed the polio-free certification in 2016 when a case of polio was reported in the North East region (Borno), one of the areas affected by conflict and insecurity.[18] However, it has been three years since the last case of the wild poliovirus was identified in Nigeria[25] and the country was eventually declared polio-free by the WHO in June 2020.[26]

Study design and participants

Three hundred and sixty-four participants, who were part of a larger descriptive survey comprising of 1020 participants, were purposively selected to participate in this study. Participants in the larger study were recruited through emails, telephone calls and in-person

For peer review only - http://bmjopen.bmj.com/site/about/guidelines.xhtml

For peer review only

contact. These means of recruitment were used to ensure the estimated sample of participants was achieved. The research team approached potential participants through the state ministry of health, the executive secretary to the minister of health to some states (Ondo and Oyo) provided a list of health workers who participated in polio program in his state. This list includes state immunization officers, surveillance officers, technical facilitators to the immunization. For the remaining states, the research team made contact with focal persons at health institutions to recruit health workers for the survey.

This study utilized the Consolidated Framework for Implementation Research (CFIR) as an evaluation framework to identify critical challenges and contributors in the implementation of community engagement activities within the PEI in Nigeria. The CFIR is an evaluation tool for assessing implementation activities, challenges and contributors. The CFIR was selected because it is a multi-theoretical framework that has been used to synthesize research evidence from various disciplines into a consolidated framework with multiple constructs of what works across different contexts and why they work. [19, 27] The framework has 39 constructs organized into five major domains found to influence the successful implementation of innovative programs. The domains assessed include Characteristics of the innovation; Outer setting including the economic, political and social context; Inner setting including organizational culture and climate; Characteristics of individuals involved in the implementation; and Process of conducting the intervention. [27]

The participants were selected from nine states across the six geopolitical zones in Nigeria and the Federal Capital Territory. The nine states, namely Nassarawa, Borno, Kano, Sokoto, Anambra, Bayelsa, Lagos, Ondo and Oyo States and the Federal Capital Territory Abuja, were purposively selected based on the existing rapport between the research team and contact persons/ facilitators in these states. The respondents for this study were eligible to participate in the study based on their engagement in PEI activities for at least one year since its inception in 1988. The respondents included program managers, policymakers, researchers and front-line field implementers affiliated with the GPEI core partner organizations, the three tiers of the government health parastatals and academic/research institutions. The respondents were identified across the three tiers of government in Nigeria – federal, state (sub-national) and local government (district). At the federal level, key stakeholders in the PEI were recruited from the National Primary Health Care Development Agency (NPHCDA), Federal Ministry of Health and GPEI core partners group including WHO, UNICEF and Bill and Melinda Gates

For peer review only - http://bmjopen.bmj.com/site/about/guidelines.xhtml

Foundation. At the state level, Polio Eradication Initiative (PEI) managers, supervisors, team leads, and facilitators were recruited. At the local government level, front line implementers in the polio program were recruited.

Survey instrument

Development of the survey instrument was informed by activities of the PEI program, tools used by the GPEI partners to generate lessons learned from the polio program and attempts to fill gaps left from previous efforts as well as external efforts. Key GPEI program activities domains (and related implementation challenges) were identified in a literature review of GPEI literature.

The survey instrument was a structured closed-ended questionnaire with sections on demographics and participants' experiences with implementing community engagement activities. The survey instrument was pretested among 10 Resident Doctors at the University College Hospital, Ibadan by the research team to ensure that questions were properly asked and adequately answered by the research assistants and respondents, respectively. These respondents provided face content and validity for the questionnaires.

The participants' experiences with implementing community engagement activities were organized into two categories:

1. Facilitators – These were the factors that contributed to the success in the implementation of community engagement activities in the PEI, Nigeria.
2. Barriers – These were the factors that impeded the implementation of community engagement activities in the PEI, Nigeria.

For the two categories, the CFIR framework was used to inform the responses. The CFIR framework contains domains and constructs which had the potential to serve as facilitators or barriers in implementing community engagement activities. (Table 1)

Table 1: CFIR domains and associated constructs

Construct		Short Description
**Internal setting**		
**I. Intervention/PEI program characteristics**		
A	Intervention Source	Perception of key stakeholders about whether the intervention is externally or internally developed

For peer review only - http://bmjopen.bmj.com/site/about/guidelines.xhtml

B	Evidence Strength & Quality	Stakeholders' perceptions of the quality and validity of evidence supporting the belief that the intervention will have desired outcomes.
C	Relative Advantage	Stakeholders' perception of the advantage of implementing the intervention versus an alternative solution.
D	Adaptability	The degree to which an intervention can be adapted, tailored, refined, or reinvented to meet local needs.
E	Trialability	The ability to test the intervention on a small scale in the organization, and to be able to reverse course (undo implementation) if warranted.
F	Complexity	Perceived difficulty of implementation, reflected by duration, scope, radicalness, disruptiveness, centrality and intricacy and number of steps required to implement.
G	Design Quality & Packaging	Perceived excellence in how the intervention was bundled, presented, and assembled.
H	Cost	Costs of the intervention and costs associated with implementing the intervention including investment, supply, and opportunity costs.
**II.**	**Inner/Organizational settings**	
A	Structural Characteristics	The social architecture, age, maturity, and size of an organization.
B	Networks & Communications	The nature and quality of webs of social networks and the nature and quality of formal and informal communications within an organization.
C	Culture	Norms, values, and basic assumptions of a given organization.
D	Implementation Climate	The absorptive capacity for change, shared receptivity of involved individuals to an intervention, and the extent to which use of that intervention will be rewarded, supported, and expected within their organization.
E	Readiness for Implementation	Tangible and immediate indicators of organizational commitment to its decision to implement an intervention – leadership engagement and resources available.
F	Others	Other personal attributes
**III.**	**Characteristics of individuals within your organization involved in PEI activities**	
A	Knowledge & Beliefs about the Intervention	Individuals' attitudes toward and value placed on the intervention as well as familiarity with facts, truths, and principles related to the intervention.
B	Self-efficacy	Individual belief in their capabilities to execute courses of action to achieve implementation goals.

For peer review only - http://bmjopen.bmj.com/site/about/guidelines.xhtml

C	Individual Stage of Change	Characterization of the phase an individual is in, as he or she progresses toward skilled, enthusiastic, and sustained use of the intervention.
D	Individual Identification with Organization	A broad construct related to how individuals perceive the organization, and their relationship and degree of commitment with that organization
E	Other Personal Attributes	A broad construct to include other personal traits such as tolerance of ambiguity, intellectual ability, motivation, values, competence, capacity, and learning style.
**IV. Process of conducting the activities**		
A	Planning	The degree to which a scheme or method of behavior and tasks for implementing an intervention are developed in advance, and the quality of those schemes or methods.
B	Engaging	Attracting and involving appropriate individuals in the implementation and use of the intervention through a combined strategy of social marketing, education, role modeling, training, and other similar activities.
C	Executing	Carrying out or accomplishing the implementation according to plan.
D	Reflecting & Evaluating	Quantitative and qualitative feedback about the progress and quality of implementation accompanied with regular personal and team debriefing about progress and experience.
**V.**	**External/Outer setting**	
A	Political environment	Lawmaker support, political climate accepting of polio eradication activities, and political structure to conducive to coordinated action
B	Economic environment	Sufficient revenue sources/base to fund activities and/or maintain system developments
C	Social environment	Social norms around immunization, accepting communities in which polio eradication activities were implemented
D	Technological environment	Infrastructure or technological advances outside of the organization
E	Other environment	Environment where activity was implemented was prohibitive and did not contribute to the success of polio eradication, including the global climate and ineffective cross-organizational collaboration

1 *Adapted from a study [16] on Fostering implementation of health services research findings*
2 *into practice: A Consolidated Framework for advancing Implementations Science*

For peer review only - http://bmjopen.bmj.com/site/about/guidelines.xhtml

Data collection procedures

The survey instrument was administered online using Qualtrics, a web-based data collection and analysis platform. The self-administered survey was in English language. The Qualtrics link was sent via email to potential respondents with instruction to respond within two weeks. Subsequently, the research team sent an email as a reminder to the potential respondents two weeks after the initial contact. The survey was conducted between September 2018 and January 2019.

Potential participants who had difficulty in participating online either as a result of a busy schedule or technical difficulties had the option of a face-to-face interview or telephone interviews. These interviews were conducted by research assistants who had at least a Bachelor's degree. All the survey data were uploaded by a research assistant on the Qualtrics platform.

Data analysis

Data were first exported from the Qualtrics platform into SPSS statistical software package, version 20. Data were then cleaned and frequencies were used to summarize the demographic characteristics. Domain analysis of the CFIR was conducted to the highest occurring factor that contributes/and impedes successful community engagement. This was further broken down into internal and external domains contributors. While for barriers to community engagement. CFIR domain analysis was conducted to identify the most significant domain and further analysis was conducted on construct level to gain more insight into these barriers.

Patient and public involvement

Patients and/or the public were not involved in the design, or conduct, or reporting, or dissemination plans of this research.

Ethical considerations

Ethical approval for this study was obtained from the National Health Research Ethics Committee (Approval Number: NHREC/01/01/2007-6/07/2018). Written informed consent was obtained from the study participant who participated in the face-to-face interviews while verbal consent was obtained from participants interviewed via the telephone after the objectives and rationale of the study had been explained. Participants who provided their responses via

For peer review only

For peer review only - http://bmjopen.bmj.com/site/about/guidelines.xhtml

the Qualtrics platform signed their consent electronically after a detailed description of the study and its rationale was provided. Participants were informed that their responses were anonymous unless they indicated that they would like to be contacted for follow-up and dissemination of survey results. They were assured that their information will remain confidential and will not be shared beyond the research team without their consent.

Results

Socio-demographic characteristics

About 249 (68.4%) participants had been engaged in the Nigerian PEI for less than 10 years. The respondents had been engaged at the various levels of implementation of PEI activities; More than half (57.4%) of the respondents worked at the subdistrict/local government level. The most common roles included team supervisors (46.9%) and front-line health workers (30.8%). Additional socio-demographic characteristics of survey participants are presented in Table 2.

Table 2: Socio-demographics characteristics of respondents

Respondents characteristics	n(%)
**Work experience (years)**	
<10	249(68.4)
10-19	96(26.4)
≥20	19(5.2)
**Level of involvement***	
District	209(57.4)
State	168(46.2)
Subdistrict/Local Government	120(32.9)
National	64(17.6)
Global	21(5.8)
**Affiliated organization***	
Government agencies	348(96.6)
Other GPEI Partners	234(64.3)
UNICEF	147(40.4)
Implementing partners	163(44.8)
Global NGO	20(5.5)
Academic research	5(1.4)
**Roles with the PEI program in Nigeria***	
Team supervisor	171(46.9)
Front line health worker	112(30.8)
Monitoring and oversight board	78(21.4)
Strategy committee/management group	57(15.6)
Program officer	37(10.2)
Surveillance officer	35(9.6)
EPI manager	32(8.8)
Program manager	25(6.9)

For peer review only - http://bmjopen.bmj.com/site/about/guidelines.xhtml

Policymaker	12(3.3)
Researcher	7(1.9)
Country project lead	6(1.6)
Others**	101(27.7)

**Multiple response questions; **Others include: Consultant, Cluster Coordinator, Data Analysis Officer, Medical Officer of Health*

Factors that contributed to the successful implementation of community engagement activities in the PEI, Nigeria

A total of 45.0% of the participants, reported that the process of conducting the PEI program (for instance, planning of stakeholder engagement, engagement of appropriate stakeholder, executing the activities as planned and monitoring engagement outcomes against the stated objectives) was the most important internal contributor to implementing community engagement activities in the community. This was followed by other internal factors 24.0% including characteristics of individuals within the organization involved in the PEI activities, awareness of benefits of community engagement, health workers enthusiasm and sustained support throughout the stages of the engagement and commitment to the organization and PEI program characteristics 21.0% including the positive perception of quality and effectiveness of community engagement activities, the adaptability of the strategy to local context, ease of implementation of the strategy, and minimal challenges encountered during implementation (see Figure 1).

The most frequently mentioned external contributor to the community engagement activities was the social environment, 56.0%, in which the community engagement activity was implemented (particularly sociocultural beliefs around immunization). Also, the political factors (stakeholders' and political support) within the communities 26.0% was a commonly mentioned external contributor. The economic factors, 16.0%, were also mentioned as contributors to community engagement activities (Figure 2).

Factors that challenge the implementation of community engagement activities in the PEI, Nigeria

The external environment (50%) such as stakeholders' lack of interest, poor funding mechanisms and norms that hinder immunization campaigns, was the most implicated factor in the challenges to community engagement activities. This is followed by the process of conducting the community engagement activities (23.1%) and the characteristics of people

For peer review only - http://bmjopen.bmj.com/site/about/guidelines.xhtml

involved in the polio eradication activities (12.4%) such as lack of awareness of benefits of the vaccine, poor knowledge of principles of vaccination (see Figure 3).

The respondents indicated that within the external environment domain, the economic environment (35.7%) such as funding mechanism and the social environment (32.9%) such as sociocultural beliefs about vaccination, were the most frequent external challenges to community engagement. Other challenges highlighted were the political environment (26.9%) such as political will and set priorities, and the technological environment (9.3%) which includes access to technology. The global climate and ineffective cross-organizational collaborations were mentioned by 29.1% of the respondents as challenging factors in the category of other environments.

As shown in Table 3, of all the processes involved in conducting PEI program activities, the process of conducting community engagement (46.4%) such as types of activities and stakeholders involved and how the activities were organized was the most important challenge to implementing community engagement strategies. This was closely followed by the execution phase (44.0%), the planning phase (35.7%), and the reflection phase (27.4%).

In the domain exploring the characteristics of individuals involved in the PEI activities within the organization, the individual's knowledge about the importance of community engagement and belief that community engagement may not be effective (62.2%) was the most reported challenge in terms of effectively engaging community members during the implementation of PEI activities. Other factors identified in this category were the individual's perception about and degree of commitment to the PEI (28.0%), the individual's stage of change (26.8%), self-efficacy (13.4%), and other personal attributes (4.9%). (Table 3)

According to respondents, the level of readiness for implementation within the PEI organization construct (46.7%) was the most frequently recorded challenge in the domain of conducting Intervention/PEI program characteristics. This was closely followed by the level of networking and communication within the organization (35.0%). The culture, implementation climate and structural characteristics within the PEI organization were the least reported challenges to community engagement (28.3%, 26.7% and 18.3% respectively) (Table 3).

For peer review only - http://bmjopen.bmj.com/site/about/guidelines.xhtml

Table 3: Categories of factors that impede the implementation of community engagement activities*

**Factors**	**Categories**	**n (%)***
External Environment	Economic	65(35.7)
	Social	60(32.9)
	Political	49(26.9)
	Technological	17(9.3)
	Other environments	53(29.1)
Processes involved in conducting PEI program activities	Engagement phase	39(46.4)
	Execution phase	37(44.0)
	Planning phase	30(35.7)
	Reflection phase	23(27.4)
Intervention/PEI program characteristics	Intervention source	17(37.8)
	Adaptability of the program to the local context	17(37.8)
	Cost	14(31.1)
	Evidence strength and relative advantgae	12(26.7)
	Relative advantage	12(26.7)
	Complexity	10(22.2)
	Design quality	9(20.0)
	Trialability	5(11.1)
	Level of readiness for implementation	28(46.7)
Inner/Organizational settings	Networks and communication	21(35.0)
	Culture	17(28.3)
	Implementation of climate	16(26.7)
	Structural charateristics	11(18.3)
Characteristics of individuals involved within the organisation	Individual's knowledge and beliefs about the activity	51(62.2)
	Individual's identification with the organization	23(28.0)
	The individual's stage of change	22(26.8)
	Self-efficacy	11(13.4)
	Other personal attributes	4(4.9)

**Multiple responses.*

For peer review only

For peer review only - http://bmjopen.bmj.com/site/about/guidelines.xhtml

Discussion

This paper identified the process of conducting community engagement activities (such as planning of stakeholder engagement, engagement of appropriate stakeholder, executing the activities as planned and monitoring engagement outcomes against intended objectives) as a major contributor to the success of community engagement strategy under the PEI program in Nigeria. Characteristics of individuals (for instance awareness of benefits of the community engagement, health workers enthusiastic and sustained support throughout the stages of the community engagement and commitment to the organization) implementing the activities were identified as another contributor. While the external environment, especially the economic and social environment (for example, sociocultural beliefs that hinder immunization such as family socioeconomic status, mothers literacy level, husbands permission before immunization, religious beliefs about vaccines, rumors and misconception surrounding vaccines, side effects of vaccination), served as a major barrier to community engagement. Hence, when properly harnessed, the social environment can contribute to the success of the community engagement goals but if neglected can hinder the successful implementation of a community engagement strategy, just like many authors have reported.[28, 29]

To harness these factors (process of conducting activities, individual characteristics, socio and economic environment), it is necessary to think about how readily modifiable these factors are. The process of conducting community engagement is a readily modifiable factor as it has evolved into a strategy that is led by the community members to ensure sustainability and ownership of the engagement process. While the characteristics of the PEI workforce have been modified over the years by equipping them with skills and abilities to provide sustained support to community engagement,[20] the sociocultural realities of a community are not readily modifiable. The PEI has successfully highlighted aspects of the culture that supports vaccination and addresses myths, beliefs and misconceptions that hinder participating in the immunization exercises.[30] Therefore, finding a balance between the positives and negatives of any community's culture should be the goal of community engagement activities and not modifying the community's culture.

In the *internal setting,* there is an interplay of vital factors such as the process of conducting activities and characteristics of individuals within the organization. According to Damschroder and colleagues, essential components for the success of community engagement activities include the process of conducting activities that involved the planning of the activities, the

For peer review only - http://bmjopen.bmj.com/site/about/guidelines.xhtml

mode of engagement and the point of entry into the community.[19] An instance of this interplay is an unmotivated health worker given a poorly planned program to implement, this increases the possibility of failure in implementation failure. Other essential considerations for successful community engagement were: the characteristics of individuals to involve and how the community engagement is executed, monitored and evaluated.[19] The polio program has documented the importance of measuring staff performance for the accountability framework and how it plays a role in achieving effective acute flaccid paralysis (AFP) surveillance. Therefore, it is believed that harnessing staff performance which falls in the category of characteristics of individuals involved for community engagement will show similarly positive results.[31]

Based on the individuals involved in the program, rreflections on Africa's polio campaigns show, that any initiative is only as good as the quality of the human resource engaging with the community.[32] The PEI focused on local champions called the volunteer community mobilizers who advocate and align with the goal of the program. They were purposeful in addressing community challenges (non-compliant households, vaccine-rejection cases, missed children) in Northern Nigeria.[33] Engagement of these individuals in the polio programs, including religious and traditional leaders has aided the program to address their misconceptions about the benefits of the polio program and improving their self-efficacy to share information on polio immunization has increased a sense of ownership of the polio program which was the primary goal of community engagement, encouraged the participation of religious and traditional leaders in the program and developed social networks within the community will be available to work on routine immunization and other health services as part of the polio legacy.[6]

The *external setting,* the social environment was identified as the most influential factor in supporting as well as undermining the community engagement process. In line with the submissions of several authors,[28, 29] the unique context, culture, beliefs, modes of communication of any community could be a threat or facilitator of community participation in any program. In the case of the polio program in the northern part of Nigeria, the culture of preventing visitors contact with their wives, beliefs about the content of the polio vaccines, their nomadic way of life and their social norms about immunization activities negatively affected the coverage rates in these areas.[13, 34, 35] However, engagement of the community

For peer review only - http://bmjopen.bmj.com/site/about/guidelines.xhtml

members resulted in the identification of the problems and proffering of mutually acceptable solutions.[36]

This resistance to immunization due to misconceptions and cultural beliefs in northern Nigeria was not as pronounced in the other parts of the country.[13, 34] This shows a sharp contrast between the northern and the southern regions of the country.[13] In essence, this shows that for health interventions to be successful, there should be some measure of contextual basis to its implementation. The sociocultural context of communities should be considered and extensive dialogue carried out to ensure communities are adequately represented and take ownership of the program. Similar to these, findings from previous studies on implementation research have also emphasized the importance of an intervention being contextual and flexible enough to be tailored to fit different environments.[27, 37]

This study also highlighted another external factor, the political environment of the community. The PEI program in Nigeria has over the years emphasized the importance of the political context in the success of any strategy both at the national scale and down to the grass-root level. For the PEI to succeed, it was important to win over political factions to increase the political will of the community representatives, avoid political interference in its activities so to make sure all community members benefit from the program regardless of political factions.[30, 38] Therefore, in addition to understanding the social context of the community, it is necessary that any community engagement seeks to understand the politics within the community, the various political factions with their beliefs and grievances. Most importantly, however, implementers must strive to remain politically neutral, to ensure that no section of the community is unrepresented due to their political affiliations.

The economic environment which involved the availability of sufficient revenues to fund immunization activities and the primary health care system were the most influential external environmental factor considered by implementers of community engagement activities. For the polio program, the Interagency Coordinating Committee (ICC) and other GPEI partners responsible for providing considerable funds to the program. These funds were used to set up 16 fully-equipped laboratories to process stool and environmental samples both for the polio program and other vaccine-preventable diseases and support the primary health system to deliver other maternal and child health interventions. The provision of health infrastructures is one of the legacies of the PEI.[39] Consequently, there have been adequate funds for the

For peer review only - http://bmjopen.bmj.com/site/about/guidelines.xhtml

successful implementation of the polio program and the support community engagement in various communities for the success of their agenda. This factor shows that engaging community members goes beyond understanding the context of the community or just advocating for increased political will. It also involves funding to recruit community members as volunteers, to provide stipends or provide logistics support to religious and traditional leaders when they attend dialogue meeting or even providing incentives to caregivers. Therefore, future program implementers should budget for the funding for community engagement activities and the sustainability of those funding for these activities even after the achievement of the project goals. This finding is corroborated by Andrus who highlighted the role of the funds in the success of programs by stating that a constant demotivator for polio personnel across endemic countries has constantly been the lack of financial incentives.[33] While Bigna[24] summarized it in his words, "*Money is the nerve of war, so, going to war against wild poliovirus will require sufficient funding to destroy it*".

Conclusion

The CFIR revealed specific contextual factors that influence the effectiveness of community engagement implementation. The *process of conducting the activities and the external environment* (economic, social and political) were very critical from this study. Program implementers should collaborate transparently with the community, foster trust and maintain a relationship with the community. These findings contribute to implementation research literature supporting the use of the CFIR in displaying constructs and domains that influence health innovation implementation. These findings can be used by policymakers and researchers to advocate for effective ways to implement community engagement activities.

Disclaimer

The funders did not have any role in study design, data collection and analysis, decision to publish, or preparation of the manuscript.

Patient consent for publication

Not required.

Contributorship statement

The research was conceived and designed by ETA, OA, AK and OOA, while data collection and analysis were implemented by ETO, OOA, AA, CN and CB. This manuscript was conceived by OOA, AA and ETO. The initial draft was developed by AA, CB and CN and

For peer review only - http://bmjopen.bmj.com/site/about/guidelines.xhtml

reviewed by OOA, AAF, AK, OA and ETO. The final version of the manuscript was approved by OOA, AA, CB, CN, AK, AAF, OA and ETA.

Competing interests

None declared

Funding

This study was funded by the Bill and Melinda Gates Foundation (Grant/Award Number: OPP1178578). The funder did not play any role in writing the protocol, interpreting the data, or in writing this manuscript. The funder provided and coordinated external peer-review for the study proposal. Publication costs were funded by the Bill and Melinda Gates Foundation.

Availability of data and materials

The data used and/or analysed in this study are available from the corresponding author on request.

Abbreviations

CFIR: Consolidated Framework for Implementation Research

CGPP: CORE Group Partners Project

GPEI: Global Polio Eradication Initiative

NPHCDA: National Primary Health care Development Agency

PEI: Polio Eradication Initiative

STRIPE: Synthesis and Translation of Research and Innovations from Polio Eradication

VCM: Volunteer Community Mobilizers

References

1. Callaghan KA. Establishing Community-Based Primary Health Care. Community-Based Health Interventions in an Institutional Context: Springer; 2019. p. 23-38.
2. Alves OMA, Moreira JP, Santos PC. Developing community partnerships for primary healthcare: An integrative review on management challenges. International Journal of Healthcare Management. 2020:1-19.

For peer review only - http://bmjopen.bmj.com/site/about/guidelines.xhtml

3. Hammanyero KI, Bawa S, Braka F, Bassey BE, Fatiregun A, Warigon C, et al. Lessons learnt from implementing community engagement interventions in mobile hard-to-reach (HTR) projects in Nigeria, 2014–2015. BMC Public Health. 2018; 18:1306.
4. World Health Organization, Global Polio Eradication Initiative. Polio endgame strategy 2019-2023: eradication, integration, certification and containment: World Health Organization2019.
5. Habib MA, Soofi S, Cousens S, Anwar S, ul Haque N, Ahmed I, et al. Community engagement and integrated health and polio immunisation campaigns in conflict-affected areas of Pakistan: a cluster randomised controlled trial. The Lancet Global Health. 2017; 5:e593-e603.
6. McArthur-Lloyd A, McKenzie A, Findley SE, Green C, Adamu F. Community engagement, routine immunization, and the polio legacy in northern Nigeria. Global Health Communication. 2016; 2:1-10.
7. Nasir S-G, Aliyu G, Ya'u I, Gadanya M, Mohammad M, Zubair M, et al. From intense rejection to advocacy: How Muslim clerics were engaged in a polio eradication initiative in Northern Nigeria. PLoS Med. 2014; 11:e1001687.
8. Fekadu L, Okeibunor J, Nsubuga P, Kipela JM, Mkanda P, Mihigo R. Reaching the unreached with polio vaccine and other child survival interventions through partnership with military in Angola. Vaccine. 2016; 34:5155-8.
9. Ongwae KM, Bawa SB, Shuaib F, Braka F, Corkum M, Isa HK. Use of Dedicated mobile teams and polio volunteer community mobilizers to increase access to zero-dose oral poliovirus vaccine and routine childhood immunizations in settlements at high risk for polio transmission in northern Nigeria. The Journal of infectious diseases. 2017; 216:S267-S72.
10. Setswe G, Witthuhn J. Community engagement in the introduction and implementation of the National Health Insurance in South Africa. Journal of Public Health in Africa. 2013; 4.
11. Dalhatu S, Ghani NA, Bozkurt V. A systematic review on factors affecting community participation towards polio immunization in Nigeria. Mediterranean Journal of Social Sciences. 2015; 6:407-.
12. Van Den Ent MM, Swift RD, Anaokar S, Hegg LA, Eggers R, Cochi SL. Contribution of global polio eradication initiative–funded personnel to the strengthening of routine immunization programs in the 10 focus countries of the polio eradication and endgame strategic plan. The Journal of Infectious Diseases. 2017; 216:S244-S9.

For peer review only - http://bmjopen.bmj.com/site/about/guidelines.xhtml

13. Ghinai I, Willott C, Dadari I, Larson HJ. Listening to the rumours: What the northern Nigeria polio vaccine boycott can tell us ten years on. Global public health. 2013; 8:1138-50.
14. Jegede AS. What led to the Nigerian boycott of the polio vaccination campaign? PLoS Med. 2007; 4:e73.
15. Cochi SL, Freeman A, Guirguis S, Jafari H, Aylward B. Global polio eradication initiative: lessons learned and legacy. The Journal of infectious diseases. 2014; 210:S540-S6.
16. Cochi SL, Hegg L, Kaur A, Pandak C, Jafari H. The global polio eradication initiative: progress, lessons learned, and polio legacy transition planning. Health Affairs. 2016; 35:277-83.
17. Aylward RB, Acharya A, England S, Agocs M, Linkins J. Global health goals: lessons from the worldwide effort to eradicate poliomyelitis. The Lancet. 2003; 362:909-14.
18. Nasir UN, Bandyopadhyay AS, Montagnani F, Akite JE, Mungu EB, Uche IV, et al. Polio elimination in Nigeria: A review. Human Vaccines & Immunotherapeutics. 2016; 12:658-
19. Damschroder LJ, Aron DC, Keith RE, Kirsh SR, Alexander JA, Lowery JC. Fostering implementation of health services research findings into practice: a consolidated framework for advancing implementation science. Implementation science. 2009; 4:1-15.
20. Alonge O, Neel A, Kalbarczyk A, Peters M, Mahendradhata Y, Sarker M, et al. Synthesis and translation of research and innovations from polio eradication (STRIPE): Initial findings from a global mixed methods study. BMC Public Health. 2020; 20.
21. CIA. The World Factbook. Central Intelligence Agency. 2012.
22. Renne E. Perspectives on polio and immunization in Northern Nigeria. Social science & medicine. 2006; 63:1857-69.
23. Hamisu AW, Johnson TM, Craig K, Mkanda P, Banda R, Tegegne SG, et al. Strategies for improving polio surveillance performance in the security-challenged Nigerian States of Adamawa, Borno, and Yobe during 2009–2014. The Journal of infectious diseases. 2016; 213:S136-S9.
24. Bigna JJR. Polio eradication efforts in regions of geopolitical strife: the Boko Haram threat to efforts in sub-Saharan Africa. African Health Sciences. 2016; 16:584-7.

For peer review only - http://bmjopen.bmj.com/site/about/guidelines.xhtml

25. Abimbola S, Malik AU, Mansoor GF. The final push for polio eradication: addressing the challenge of violence in Afghanistan, Pakistan, and Nigeria. PLoS Med. 2013; 10:e1001529.
26. NAN. WHO declares Nigeria Polio free. The Guardian. 2020 19 June 2020.
27. Saluja S, Silverstein A, Mukhopadhyay S, Lin Y, Raykar N, Keshavjee S, et al. Using the Consolidated Framework for Implementation Research to implement and evaluate national surgical planning. BMJ global health. 2017; 2.
28. Pell CL, Adhikari B, Myo Thwin M, Kajeechiwa L, Nosten S, Nosten FH, et al. Community engagement, social context and coverage of mass anti-malarial administration: Comparative findings from multi-site research in the Greater Mekong sub-Region. PLoS One. 2019; 14:e0214280.
29. Sahan K, Pell C, Smithuis F, Phyo AK, Maung SM, Indrasuta C, et al. Community engagement and the social context of targeted malaria treatment: a qualitative study in Kayin (Karen) State, Myanmar. Malaria journal. 2017; 16:1-10.
30. Usman S, Bologna L, Stamidis KV. The CORE Group Partners Project in North East Nigeria: community engagement strategies to combat skepticism and build trust for vaccine acceptance. The American journal of tropical medicine and hygiene. 2019; 101:68-73.
31. Kassahun A, Braka F, Gallagher K, Gebriel AW, Nsubuga P, M'pele-Kilebou P. Introducing an accountability framework for polio eradication in Ethiopia: results from the first year of implementation 2014-2015. The Pan African Medical Journal. 2017; 27.
32. Kumar P, Khan A. Human resource management in primary health care system. Health and Population–Perspectives and Issues. 2013; 36:66-76.
33. Andrus JK, Perry HB. Community engagement, ownership, and civil society organizations in polio eradication. The American journal of tropical medicine and hygiene. 2019; 101:1.
34. Yahya M. Polio vaccines—"no thank you!" barriers to polio eradication in Northern Nigeria. African Affairs. 2007; 106:185-204.
35. Taylor SA. Culture and behaviour in mass health interventions: lessons from the global polio eradication initiative. Critical Public Health. 2015; 25:192-204.
36. Coates EA, Waisbord S, Awale J, Solomon R, Dey R. Successful polio eradication in Uttar Pradesh, India: the pivotal contribution of the Social Mobilization Network, an NGO/UNICEF collaboration. Global Health: Science and Practice. 2013; 1:68-83.

For peer review only - http://bmjopen.bmj.com/site/about/guidelines.xhtml

37. Akinyemi O, Harris B, Kawonga M. Health system readiness for innovation scale-up: the experience of community-based distribution of injectable contraceptives in Nigeria. BMC health services research. 2019; 19:1-11.
38. Haq Z, Shaikh BT, Tran N, Hafeez A, Ghaffar A. System within systems: challenges and opportunities for the Expanded Programme on Immunisation in Pakistan. Health research policy and systems. 2019; 17:51.
39. Craig AS, Haydarov R, O'Malley H, Galway M, Dao H, Ngongo N, et al. The public health legacy of polio eradication in Africa. The Journal of Infectious Diseases. 2017; 216:S343-S50.

For peer review only

For peer review only - http://bmjopen.bmj.com/site/about/guidelines.xhtml

Figure Legend/Caption

Figure 1: Internal contributors to the successful implementation of community engagement activities

Figure 2: External contributors to the successful implementation of community engagement activities

Figure 3: Factors that impede the implementation of community engagement activities

For peer review only

For peer review only - http://bmjopen.bmj.com/site/about/guidelines.xhtml

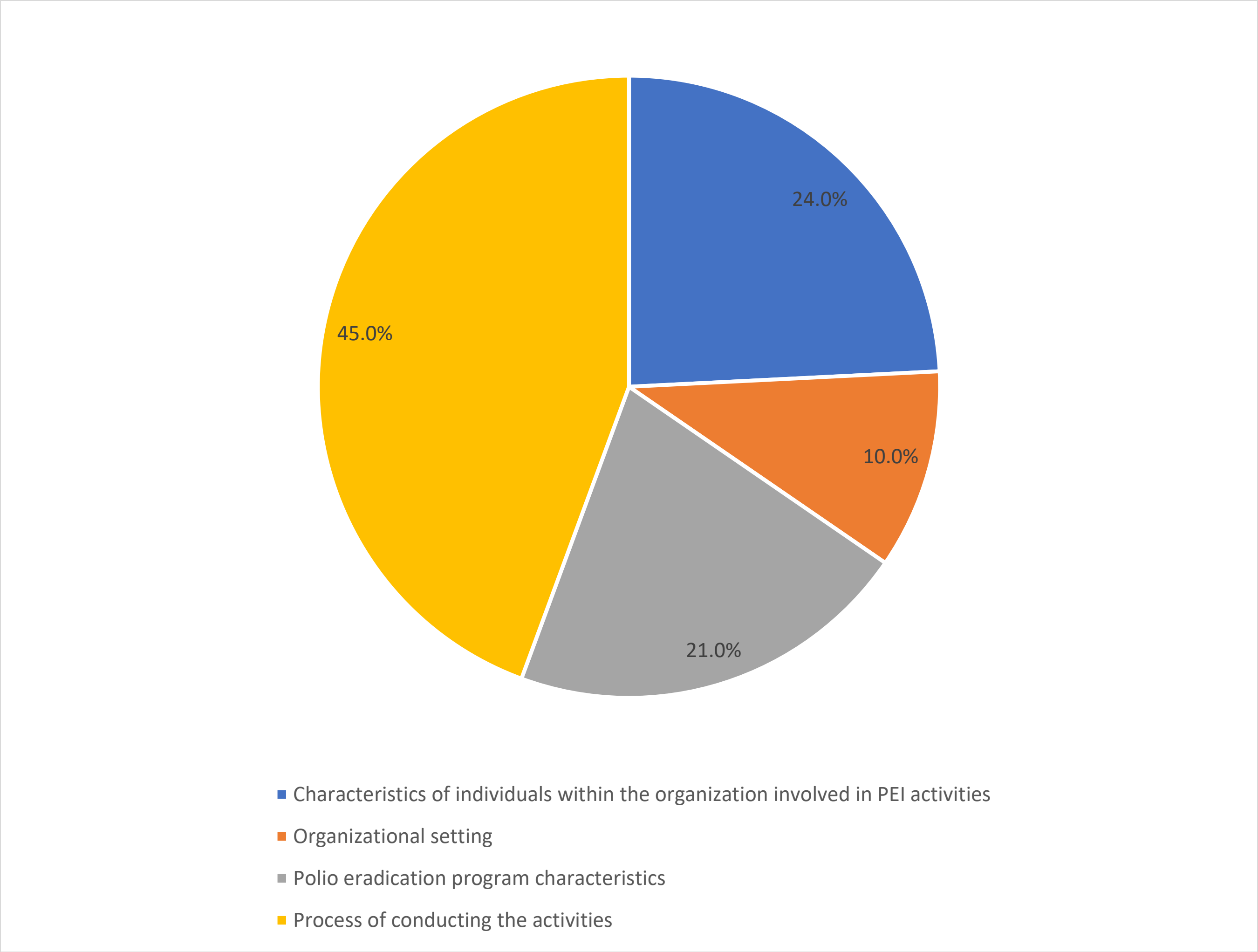

Figure 1: Internal contributors to the successful implementation of community engagement activities.

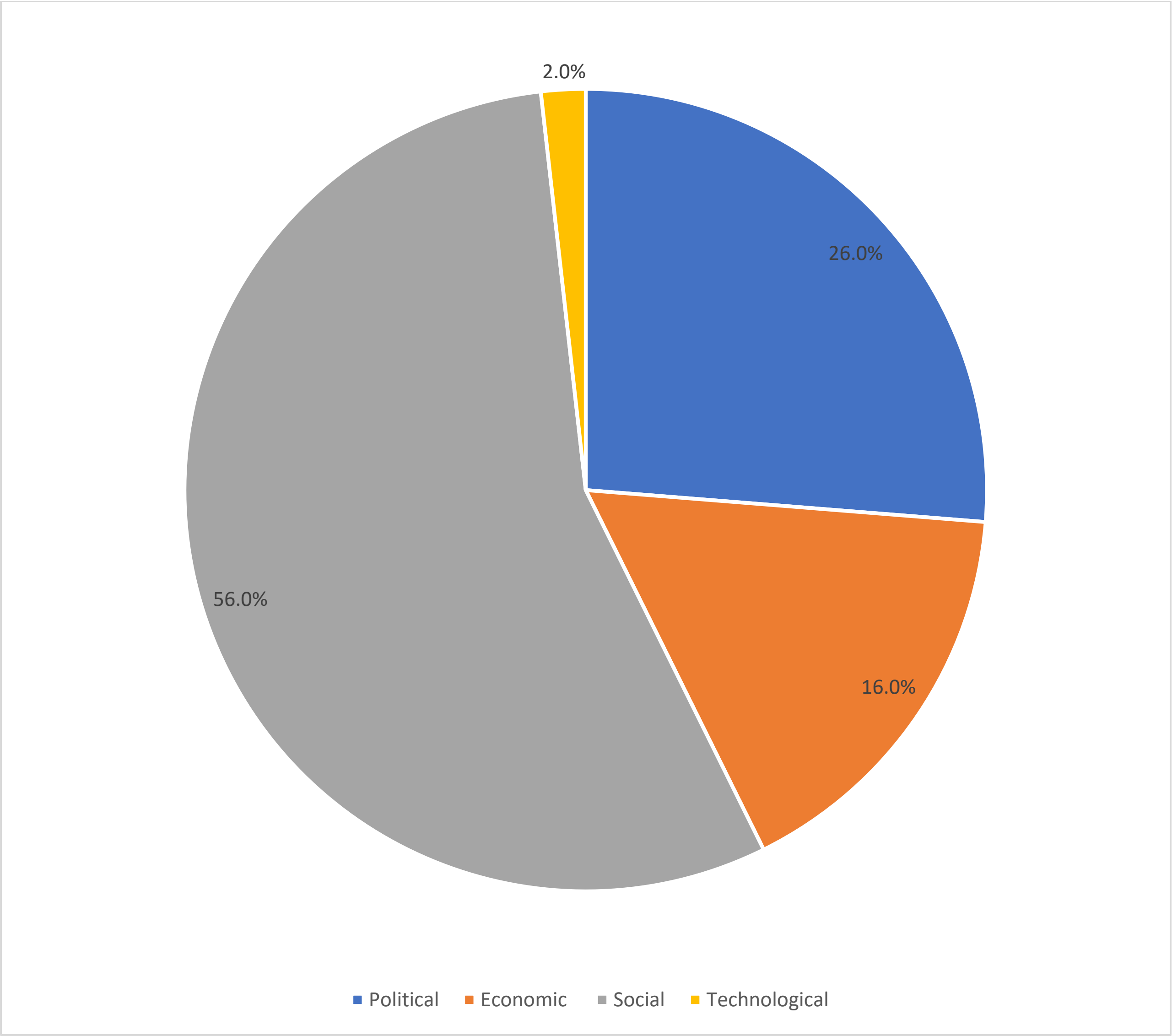

Figure 2: External contributors to the successful implementation of community engagement activities.

For peer review only - http://bmjopen.bmj.com/site/about/guidelines.xhtml

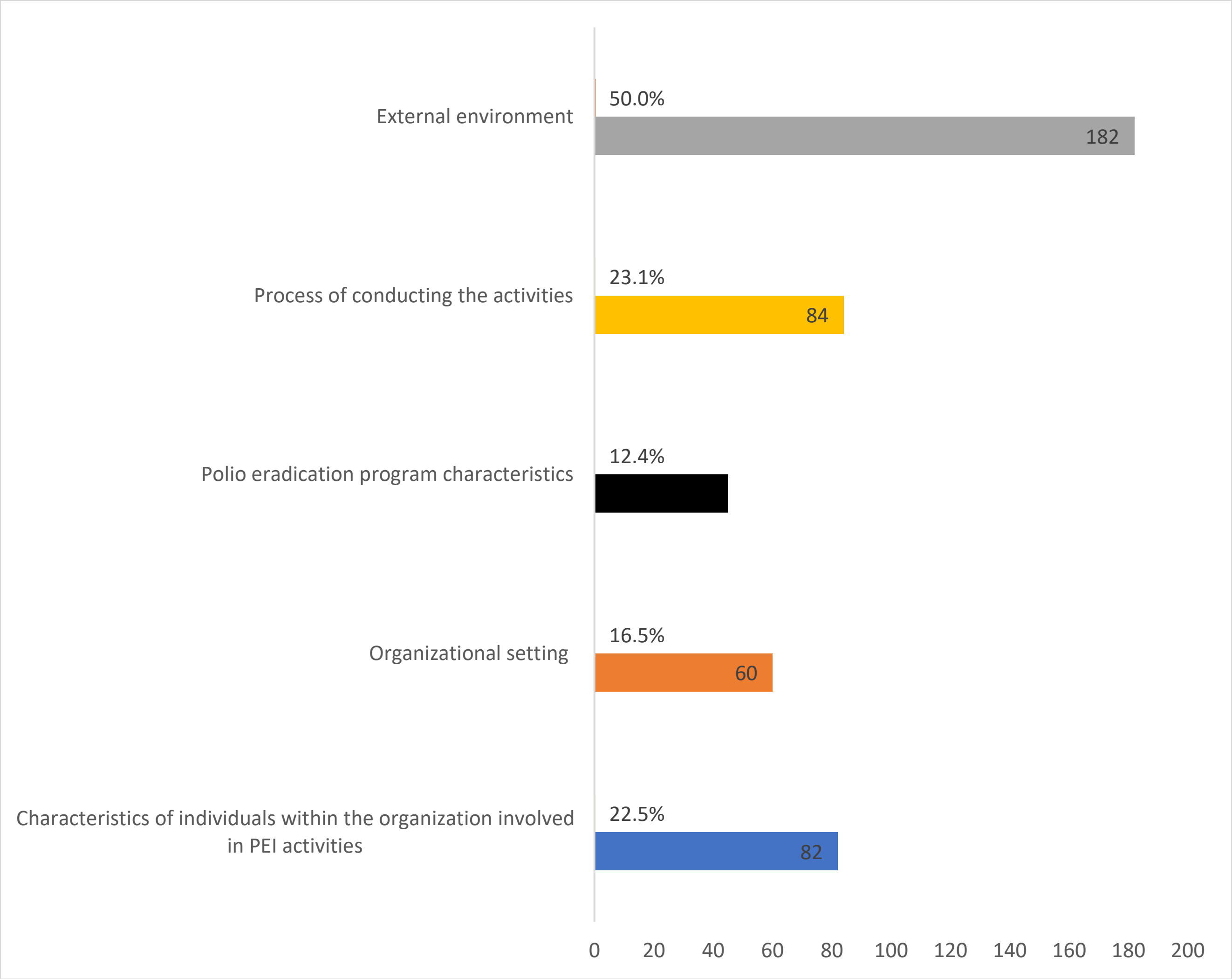

Figure 3: Factors that impede the implementation of community engagement activities.

STROBE Statement—Checklist of items that should be included in reports of ***cross-sectional studies***

	Item No	Recommendation	Page No
**Title and abstract**	1	(*a*) Indicate the study's design with a commonly used term in the title or the abstract	1
		(*b*) Provide in the abstract an informative and balanced summary of what was done and what was found	2
**Introduction**			
Background/rationale	2	Explain the scientific background and rationale for the investigation being reported	3
Objectives	3	State specific objectives, including any prespecified hypotheses	5
**Methods**			
Study design	4	Present key elements of study design early in the paper	6
Setting	5	Describe the setting, locations, and relevant dates, including periods of recruitment, exposure, follow-up, and data collection	5
Participants	6	(*a*) Give the eligibility criteria, and the sources and methods of selection of participants	6
Variables	7	Clearly define all outcomes, exposures, predictors, potential confounders, and effect modifiers. Give diagnostic criteria, if applicable	5,7
Data sources/ measurement	8*	For each variable of interest, give sources of data and details of methods of assessment (measurement). Describe comparability of assessment methods if there is more than one group	6-7, 10
Bias	9	Describe any efforts to address potential sources of bias	3
Study size	10	Explain how the study size was arrived at	6
Quantitative variables	11	Explain how quantitative variables were handled in the analyses. If applicable, describe which groupings were chosen and why	10
Statistical methods	12	(*a*) Describe all statistical methods, including those used to control for confounding	10
		(*b*) Describe any methods used to examine subgroups and interactions	10
		(*c*) Explain how missing data were addressed	10
		(*d*) If applicable, describe analytical methods taking account of sampling strategy	N/A
		(*e*) Describe any sensitivity analyses	N/A
**Results**			
Participants	13*	(a) Report numbers of individuals at each stage of study—eg numbers potentially eligible, examined for eligibility, confirmed eligible, included in the study, completing follow-up, and analysed	11-12
		(b) Give reasons for non-participation at each stage	
		(c) Consider use of a flow diagram	
Descriptive data	14*	(a) Give characteristics of study participants (eg demographic, clinical, social) and information on exposures and potential confounders	11
		(b) Indicate number of participants with missing data for each variable of interest	
Outcome data	15*	Report numbers of outcome events or summary measures	13-14

For peer review only

For peer review only - http://bmjopen.bmj.com/site/about/guidelines.xhtml

Main results	16	(*a*) Give unadjusted estimates and, if applicable, confounder-adjusted estimates and their precision (eg, 95% confidence interval). Make clear which confounders were adjusted for and why they were included	11-14
		(*b*) Report category boundaries when continuous variables were categorized	N/A
		(*c*) If relevant, consider translating estimates of relative risk into absolute risk for a meaningful time period	N/A
Other analyses	17	Report other analyses done—eg analyses of subgroups and interactions, and sensitivity analyses	N/A
**Discussion**			
Key results	18	Summarise key results with reference to study objectives	15-18
Limitations	19	Discuss limitations of the study, taking into account sources of potential bias or imprecision. Discuss both direction and magnitude of any potential bias	3
Interpretation	20	Give a cautious overall interpretation of results considering objectives, limitations, multiplicity of analyses, results from similar studies, and other relevant evidence	14-18
Generalisability	21	Discuss the generalisability (external validity) of the study results	18
**Other information**			
Funding	22	Give the source of funding and the role of the funders for the present study and, if applicable, for the original study on which the present article is based	19

N/A - Not applicable

*Give information separately for exposed and unexposed groups.

Note: An Explanation and Elaboration article discusses each checklist item and gives methodological background and published examples of transparent reporting. The STROBE checklist is best used in conjunction with this article (freely available on the Web sites of PLoS Medicine at http://www.plosmedicine.org/, Annals of Internal Medicine at http://www.annals.org/, and Epidemiology at http://www.epidem.com/). Information on the STROBE Initiative is available at www.strobe-statement.org.

For peer review only
